# Supplementary material for: Antiparasitic Ovalicin Derivatives from Pseudallescheria boydii, a Mutualistic Fungus of French Guiana Termites
Source: Molecules. 2022 Feb 10;27(4):1182. doi: 10.3390/molecules27041182 (PMC8877094; doi:10.3390/molecules27041182)
Supplement: Supplementary file 1 [file molecules-27-01182-s001.zip › molecules-1574233-supplementary.pdf]

Supplementary material for :

# Antiparasitic Ovalicin Derivatives from *Pseudallescheria boydii*, a Mutualistic Fungus of French Guiana Termites

**Jonathan Sorres[1,*], Téo Hebra[1], Nicolas Elie[1], Charlotte Leman-Loubière[1], Tatyana Grayfer[1], Philippe Grellier[2], David Touboul[1], Didier Stien[3] and Véronique Eparvier[1,*]**

1 CNRS, Institut de Chimie des Substances Naturelles, UPR 2301, Université Paris-Saclay, 91198 Gif-sur-Yvette, France; teo.hebra@cnrs.fr (T.H.); nicolas.elie@cnrs.fr (N.E.); charlotte.leman-loubiere@clarins.com (C.L.-L.); tgrayfer@gmail.com (T.G.); david.touboul@cnrs.fr (D.T.)

2 CNRS, Département RDDM, UMR 7245, Muséum National d'Histoire Naturelle CP52, 57 Rue Cuvier, 75005 Paris, France; philippe.grellier@mnhn.fr (P.G.)

3 Laboratoire de Biodiversité et Biotechnologie Microbiennes, CNRS, Sorbonne Université, Observatoire Océanologique, 66650 Banyuls-sur-Mer, France; didier.stien@cnrs.fr (D.S.)

* Correspondence: jon.sorres@gmail.com (J.S.); veronique.eparvier@cnrs.fr (V.E.)

Figure S1. $^{1}$H NMR (500 MHz, $CD_3OD$) spectrum of ovalicin (**1**) ............................................4
Figure S2. ESI-HRMS spectra of ovalicin (**1**).................................................................5
Figure S3. $^{1}$H NMR (500 MHz, $CD_3OD$) spectrum of ovalicin linoleate (**2**) ..............................6
Figure S4. $^{13}$C NMR (125 MHz, $CD_3OD$) spectrum of ovalicin linoleate (**2**) ..............................7
Figure S5. COSY NMR (500 MHz, $CD_3OD$) spectrum of ovalicin linoleate (**2**)..........................8
Figure S6. HSQC NMR spectrum of ovalicin linoleate (**2**) in $CD_3OD$ ........................................8
Figure S7. HMBC NMR spectrum of ovalicin linoleate (**2**) in $CD_3OD$........................................9
Figure S8. NOESY NMR (500 MHz, $CD_3OD$) spectrum of ovalicin linoleate (**2**) ......................9
Figure S9. ESI-HRMS spectra of **2** ...............................................................................10
Figure S10. $^{1}$H NMR (500 MHz, $CD_3OD$) spectrum of ovalicin oleate (**3**) ..............................12
Figure S11. $^{13}$C NMR (125 MHz, $CD_3OD$) spectrum of ovalicin oleate (**3**) ..............................13
Figure S12. COSY NMR (500 MHz, $CD_3OD$) spectrum of ovalicin oleate (**3**).........................13
Figure S13. HSQC NMR (500 MHz, $CD_3OD$) spectrum of ovalicin oleate (**3**).........................14
Figure S14. HMBC NMR (500 MHz, $CD_3OD$) spectrum of ovalicin oleate (**3**) ........................14
Figure S15. ESI-HRMS spectra of **3**.................................................................................15
Figure S16. $^{1}$H NMR (500 MHz, $CD_3OD$) spectrum of ovalicin stearate (**4**)............................17
Figure S17. $^{13}$C NMR (125 MHz, $CD_3OD$) spectrum of ovalicin stearate (**4**)...........................17
Figure S18. COSY (500 MHz, $CD_3OD$) spectrum of ovalicin stearate (**4**) ...............................18
Figure S19. HMQC (500 MHz, $CD_3OD$) spectrum of ovalicin stearate (**4**)...............................18
Figure S20. HMBC (500 MHz, $CD_3OD$) spectrum of ovalicin stearate (**4**) ...............................19
Figure S21. ESI-HRMS spectra of **4**.................................................................................19
Figure S22. $^{1}$H NMR (800 MHz, $CD_3OD$) spectrum of ovalicin palmitoleate (**5**) .....................20
Figure S23. ESI-HRMS spectra of **5**.................................................................................20
Figure S24. $^{1}$H NMR (800 MHz, $CD_3OD$) spectrum of demethylovalicin linoleate (**6**).............22
Figure S25. $^{13}$C NMR (200 MHz, $CD_3OD$) spectrum of demethylovalicin linoleate (**6**)............23
Figure S26. COSY (800 MHz, $CD_3OD$) spectrum of demethylovalicin linoleate (**6**) ................24
Figure S27. HSQC (800 MHz, $CD_3OD$) spectrum of demethylovalicin linoleate (**6**) ................24
Figure S28. HMBC (800 MHz, $CD_3OD$) spectrum of demethylovalicin linoleate (**6**) ...............25
Figure S29. ESI-HRMS spectra of **6**.................................................................................25
Figure S30. HRMS analyses of **2** ....................................................................................26
Figure S31. HRMS analyses of **2b** ..................................................................................27
Figure S32. HRMS analyses of **3** ....................................................................................28
Figure S33. CD spectra (MeOH) of ovalicin (**1**) and ovalicin esters (**2**–**4**). ...............................29
Figure S34. Synthesis of hemisynthetic compounds **2b**, **3b** and **4b**..........................................29
Figure S35. $^{1}$H NMR (500 MHz, $CD_3OD$) spectrum of synthetic ovalicin linoleate (**2b**)...........30
Figure S36. Comparison between $^{1}$H NMR (500 MHz, $CD_3OD$) spectra of synthetic ovalicin linoleate (**2b**) and natural Ovalicin linoleate (**2**)......................................................................30
Figure S37. $^{1}$H NMR (500 MHz, $CD_3OD$) spectrum of synthetic ovalicin oleate (**3b**) ..............31
Figure S38. Comparison between $^{1}$H NMR (500 MHz, $CD_3OD$) spectra of synthetic ovalicin oleate (**3b**) and natural ovalicin oleate (**3**). ......................................................................32
Figure S39. $^{1}$H NMR (500 MHz, $CD_3OD$) spectrum of synthetic ovalicin stearate (**4b**)............33
Figure S40. Comparison between $^{1}$H NMR (500 MHz, $CD_3OD$) spectra of synthetic ovalicin stearate (**4b**) and natural ovalicin stearate (**4**). ..................................................................33
Figure S 41. ESI-HRMS spectra of **2b** ..............................................................................34
Figure S42. ESI-HRMS spectra of **3b** ...............................................................................34
Figure S43. ESI-HRMS spectra of **4b** ...............................................................................35

Figure S44. (A) Molecular network of crude extracts of P. boydii. SNB-CN71, -CN73, -CN81, -CN85 (B) Cluster for the sodiated molecular ion of pseurotin A (C) Cluster containing the sodiated molecular ions of ovalicin (**1**) and its ester analogues. (D) Cluster of the protonated molecular ions of tyroscherin and N-methyltyrocherin. Relative quantification of each ion within the fractions are represented as a XIC area-dependent pie-chart drawing. ................................36
Figure S45. Fragmentation spectra of ovalicin fatty acids ......................................................37
Figure S46. Compounds **1** and **13** MS/MS spectra with their main fragments in bold and common neutral loss in black. ...........................................................................................................42
Figure S47. $^{1}$H NMR (800 MHz, $CD_3OD$) spectrum of **7** ........................................................43
Figure S48. COSY (800 MHz, $CD_3OD$) spectrum **7** ...............................................................43
Figure S49. HSQC (800 MHz, $CD_3OD$) spectrum of **7**...........................................................44
Figure S50. HMBC (800 MHz, $CD_3OD$) spectrum of **7**...........................................................44
Figure S51. ESI-HRMS of compound **7** .......................................................................................45

Table S1. NMR data table for ovalicin (**1**) and comparison with literature data. .........................4
Table S2. Full NMR data ovalicin linoleate (**2**) recorded in $CD_3OD$..........................................5
Table S3. Full NMR data ovalicin oleate (**3**) recorded in $CD_3OD$............................................11
Table S4. Full NMR data ovalicin stearate (**4**) recorded in $CD_3OD$ ..........................................16
Table S5. Full NMR data (500 MHz, $CD_3OD$) of demethylovalicin linoleate (**6**)......................21
Table S6. Stereochemistry of the fatty acid double bounds……………………………………..29
Table S7. Basic genomes statistics and BUSCO scores ..........................................................39
Table S8. *Pbo_Ova* biosynthetic gene cluster annotation.........................................................40
Table S9. *Pbo_Pse* biosynthetic gene cluster annotation.........................................................41
Table S10. Annotated ovalicins from *P. boydii* crude extracts, synthetized by *Pbo_Ova* biosynthetic pathway.......................................................................................................42
Table S11. Full NMR data of **7** recorded in $CD_3OD$ ................................................................45

Table S1. NMR data table for ovalicin (**1**) and comparison with literature data.

| | Experimental | Literature[1] |
|---|---|---|
| **Position** | **$\delta_H$ (*J* in Hz)[a]** | **$\delta_H$ (*J* in Hz)[b]** |
| 1 | | |
| 2 | a: 2.71, dddd (14.0, 12.1, 6.7, 1.0) | a: 2.62, ddd (12.5, 7.3, 6.7) |
| | b: 2.37, ddd (14.0, 5.6, 3.2) | b: 2.27, m |
| 3 | a: 2.48, ddd (13.6, 12.1, 5.6) | a: 2.41, dd (13.2, 5.3) |
| | b: 1.52, ddd (13.6, 6.8, 3.2) | b: 1.42, ddd (13.2, 7.3, 2.3) |
| 4 | | |
| 5 | | |
| 6 | | |
| 7 | 4.36, d (0.9) | 4.27, d (0.7) |
| 8 | 2.90, t (6.5) | 2.80, t (6.5) |
| 9 | a: 2.35, m | 2.27, m |
| | b: 2.20, m | 2.10, ddd (14.5, 7.3, 2.0) |
| 10 | 5.23, m | 5.13, tq (7.3, 1.3) |
| 11 | | |
| 12 | 1.67, brs | 1.58, s |
| 13 | 1.75, d (0.9) | 1.66, s |
| 14 | a: 3.07, d (4.4) | a: 2.97, d (4.3) |
| | b: 2.72, d (4.4) | b: 2.63, d (4.3) |
| 15 | 1.36, s | 1.26, s |
| $OCH_3$ | 3.49, s | 3.39, s |

(a) $^1H$ NMR data recorded in $CD_3OD$ at 500 MHz. (b) $^1H$ NMR data recorded in $CD_3OD$ at 270 MHz.

Reference [62]: Takamatsu, S.; Kim, Y.-P.; Komiya, T.; Sunazuka, T.; Hayashi, M.; Tanaka, H.; Komiyama, K.; Omura, S. *J. Antibiotics* **1996**, *49*, 635.

*Figure S1.* $^1H$ NMR (500 MHz, $CD_3OD$) spectrum of ovalicin (**1**)

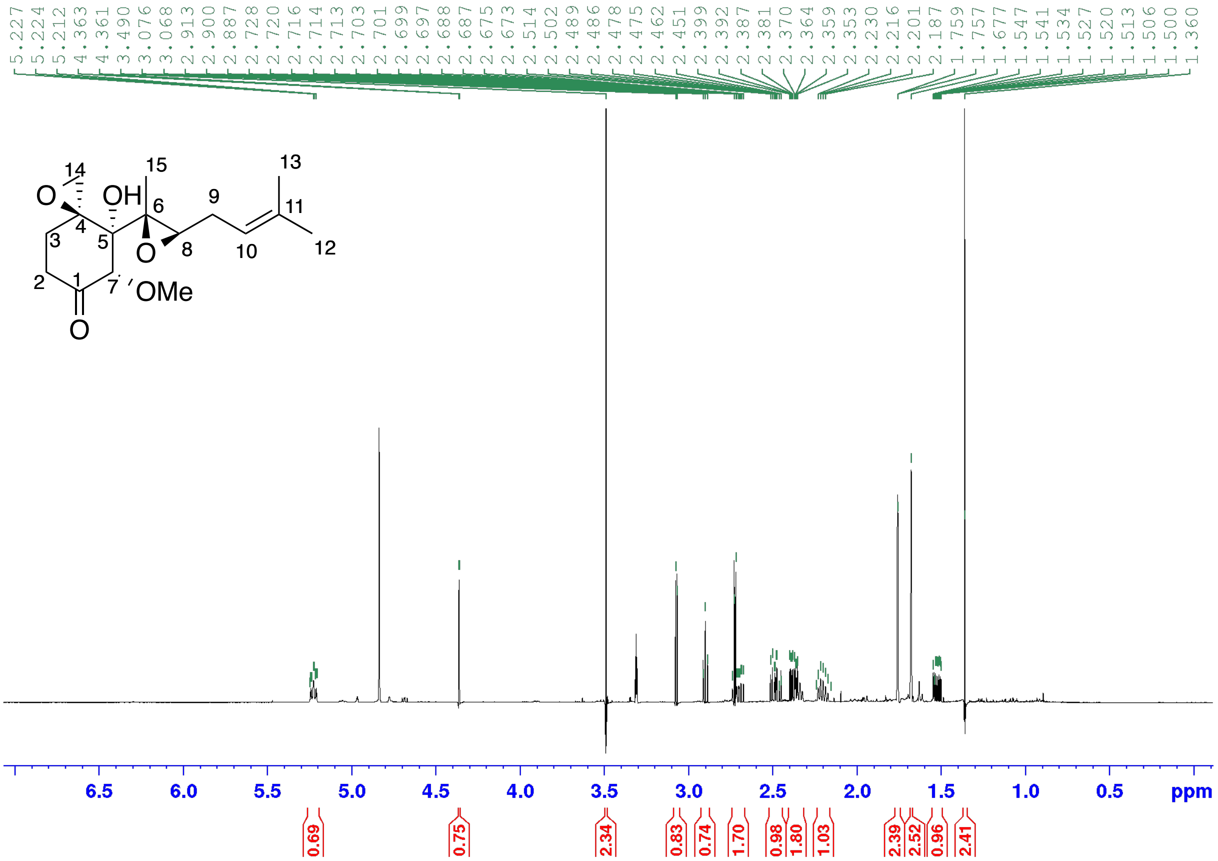

*Figure S2.: ESI-HRMS spectra of ovalicin (**1**)*

**Elemental Composition Report** Page 1 of 1

**Single Mass Analysis**
Tolerance = 10.0 PPM / DBE: min = -1.5, max = 100.0
Element prediction: Off
Number of isotope peaks used for i-FIT = 9

Monoisotopic Mass, Even Electron Ions
411 formula(e) evaluated with 3 results within limits (all results (up to 1000) for each mass)
Elements Used:
C: 1-120 H: 1-150 N: 0-10 O: 0-15
STIEN |sorres42-1 439 (2.094) Cm (437:447)
1: TOF MS ES+

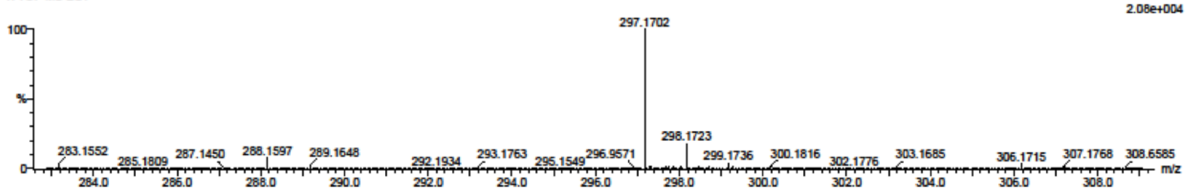


| | | | | | | | |
|---|---|---|---|---|---|---|---|
| Minimum: | | | | -1.5 | | | |
| Maximum: | | 5.0 | 10.0 | 100.0 | | | |
| Mass | Calc. Mass | mDa | PPM | DBE | i-FIT | i-FIT (Norm) | Formula |
| 297.1702 | 297.1702 | 0.0 | 0.0 | 4.5 | 777.8 | 0.1 | C16 H25 O5 |
| | 297.1675 | 2.7 | 9.1 | 5.5 | 780.3 | 2.7 | C12 H21 N6 O3 |
| | 297.1715 | -1.3 | -4.4 | 9.5 | 780.4 | 2.8 | C17 H21 N4 O |

*Table S2. Full NMR data ovalicin linoleate (**2**) recorded in $CD_3OD$*

| Position | $\delta_C$, type | $\delta_H$ (*J* in Hz) | COSY | HMBC | NOESY |
|---|---|---|---|---|---|
| 1 | 211.1, C | | | | |
| 2 | 36.5, $CH_2$ | a: 2.74, m | 2b, 3a, 3b | 1, 3 | 2b, 7 |
| | | b: 2.22, m | 2a, 3a, 3b | 1, 4, 7 | 2a |
| 3 | 32.8, $CH_2$ | a: 2.10, dd (13.6, 5.1) | 2a, 2b, 3b | 1, 4 | 3b |
| | | b: 2.01, ddd (13.6, 7.1, 2.0) | 2a, 2b, 3a | 1, 2, 4, 5 | 2a, 3a, 14b |
| 4 | 76.5, C | | | | |
| 5 | 82.8, C | | | | |
| 6 | 62.8, C | | | | |
| 7 | 86.2, CH | 4.69, s | | 1, 5, OMe | 2a, 15, OMe |
| 8 | 58.4, CH | 2.97, t (6.5) | 9a, 9b | 5, 6, 9, 10 | 14a, 14b |
| 9 | 28.4, $CH_2$ | a: 2.38, m | 8, 9b, 10 | 6, 8, 10, 11 | 8, 15 |
| | | b: 2.26, m | 8, 9a, 10 | 8, 10, 11 | 8, 15 |
| 10 | 119.8, CH | 5.28, m | 9a, 9b | 12, 13 | 12 |
| 11 | 136.1, C | | | | |
| 12 | 26.1, $CH_3$ | 1.72, brd (1.2) | 10 | 10, 11, 13 | 10 |
| 13 | 18.2, $CH_3$ | 1.68, brd (0.9) | 10 | 10, 11, 12 | |
| 14 | 69.4, $CH_2$ | a: 4.17, d (11.2) | 14b | 3, 4, 5, 1’ | 15 |
| | | b: 4.11, d (11.2) | 14a | 3, 4, 5, 1’ | 3a, 3b |
| 15 | 16.3, $CH_3$ | 1.54, s | | 5, 6, 8 | 7, 9a, 9b, OMe |
| OMe | 59.7, $CH_3$ | 3.49, s | | 7 | 7, 15 |
| 1’ | 175.5, C | | | | |
| 2’ | 35.1, $CH_2$ | 2.38, t (7.4) | 3’ | 1’, 3’, 4’ | |
| 3’ | 26.2, $CH_2$ | 1.64, m | 2’, 4’ | | |
| 4’−7’ | 30.3−30.8, $CH_2$ | 1.28, m−1.40, m | | | |
| 8’ | 28.3, $CH_2$ | 2.07, m | 7’, 9’ | | |
| 9’ | 131.0, CH | 5.36, m | 8’, 10’ | 8’ | |
| 10’ | 129.3, CH | 5.33, m | 9’, 11’ | 11’ | |
| 11’ | 26.7, $CH_2$ | 2.78, m | 10’, 12’ | 9’, 10’, 12’, 13’ | |
| 12’ | 129.2, CH | 5.33, m | 11’, 13’ | 11’ | |
| 13’ | 131.1, CH | 5.36, m | 12’, 14’ | 14’ | |
| 14’ | 28.3, $CH_2$ | 2.07, m | 13’, 15’ | | |
| 15’ | 30.3−30.8, $CH_2$ | 1.28, m−1.40, m | | | |
| 16’ | 32.8, $CH_2$ | 1.31, m | | | |
| 17’ | 23.8, $CH_2$ | 1.34, m | | | |
| 18’ | 14.6, $CH_3$ | 0.91, t (7.0) | 17’ | 16’, 17’ | |

*Figure S3.* $^1H$ *NMR (500 MHz,* $CD_3OD$*) spectrum of ovalicin linoleate (**2**)*

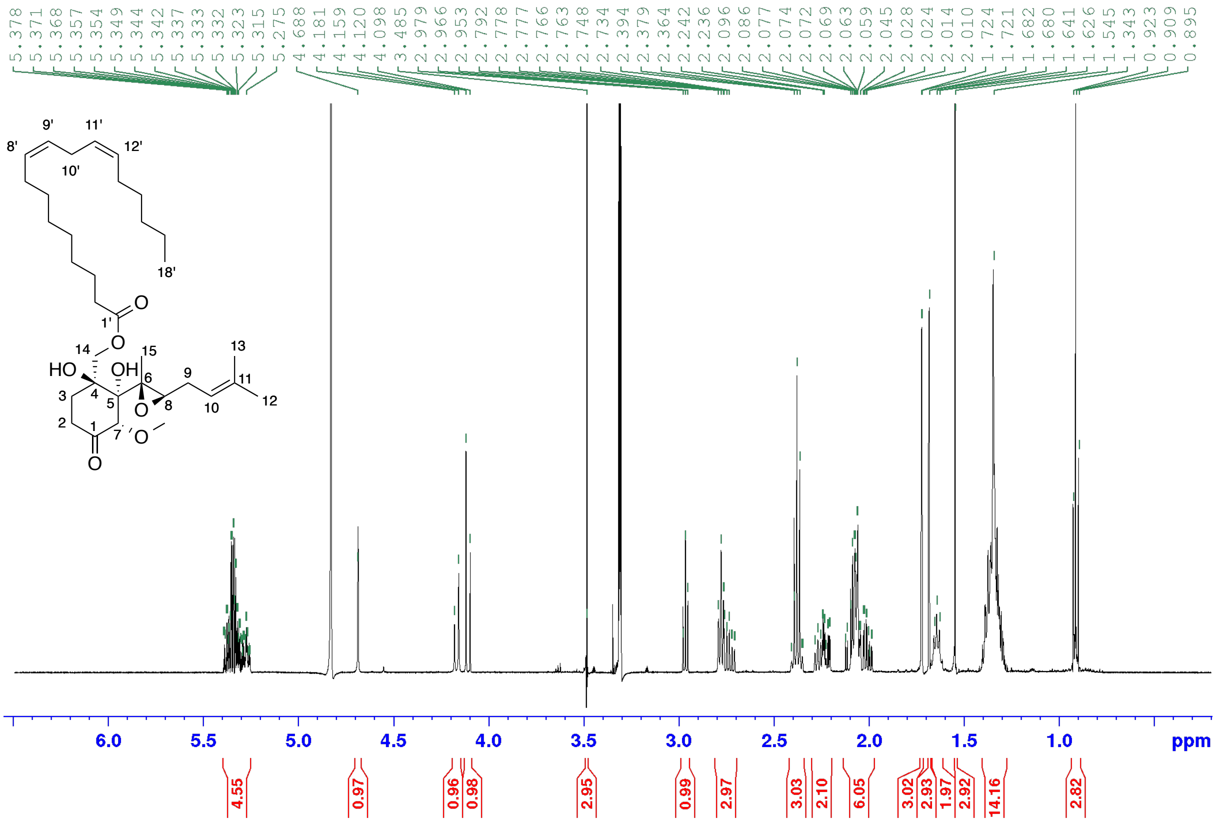

*Figure S4.* $^{13}$C NMR (125 MHz, $CD_3OD$) spectrum of ovalicin linoleate (**2**)

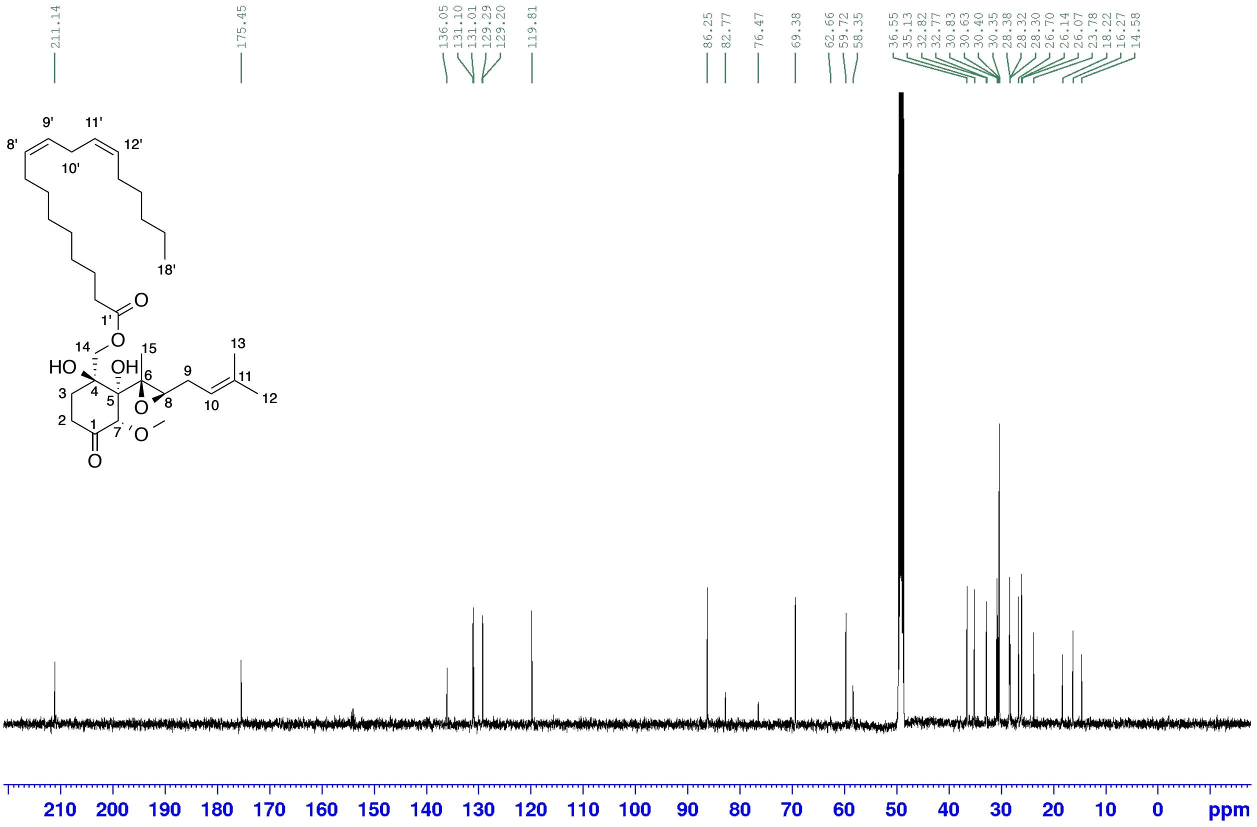

*Figure S5***.** *COSY NMR (500 MHz, $CD_3OD$) spectrum of ovalicin linoleate (**2**)*

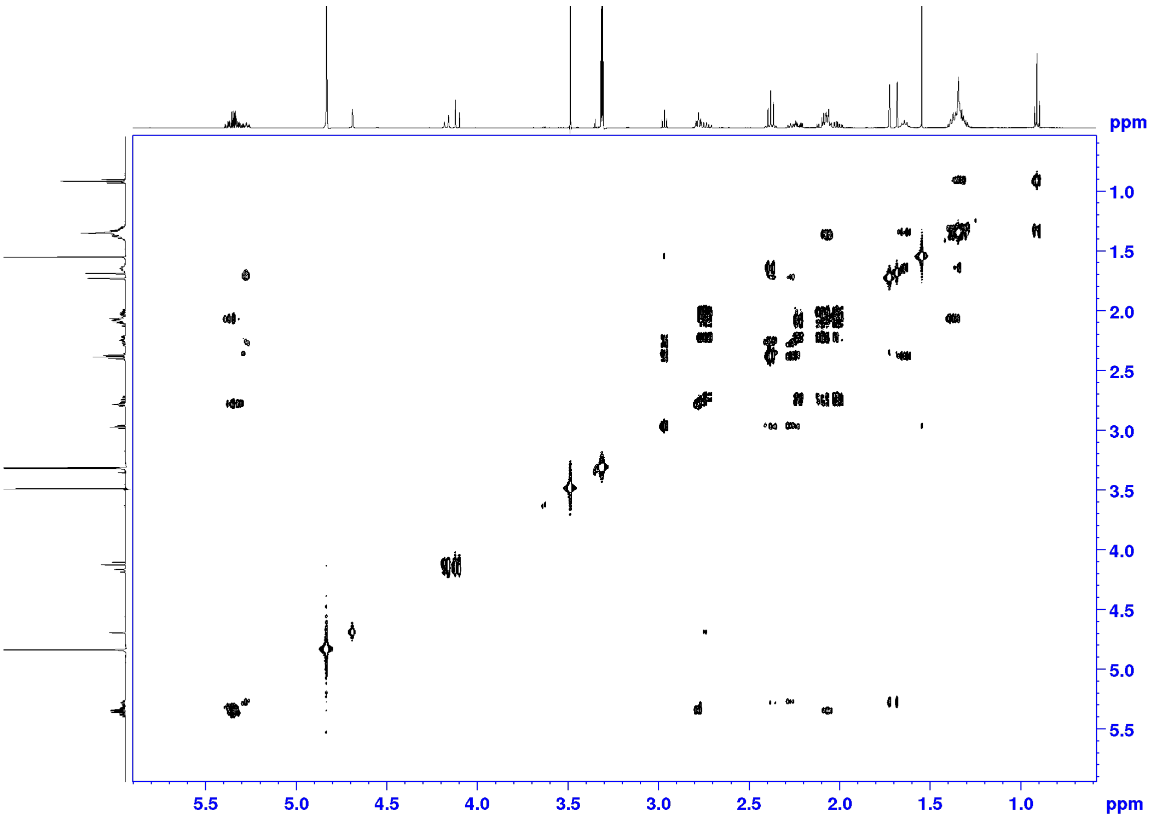


*Figure S6***.** *HSQC NMR spectrum of ovalicin linoleate (**2**) in $CD_3OD$*

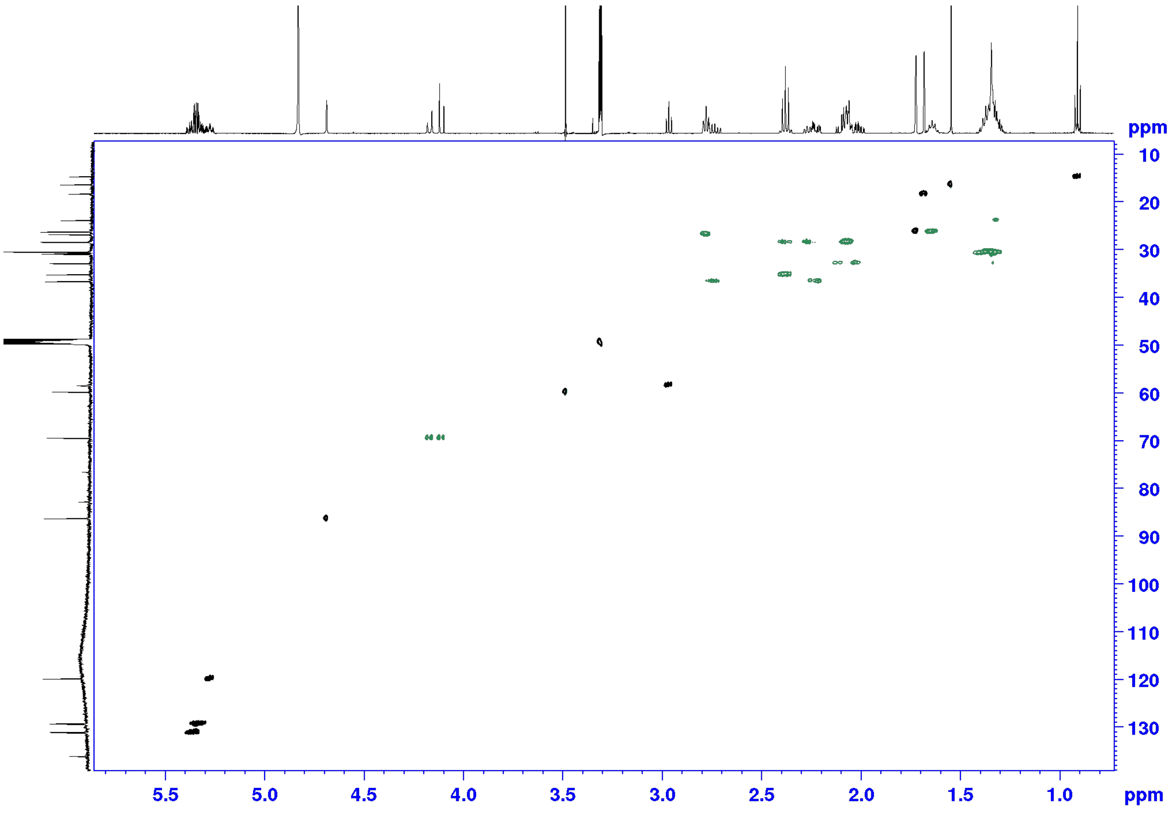

*Figure S7.* *HMBC NMR spectrum of ovalicin linoleate (**2**) in $CD_3OD$*

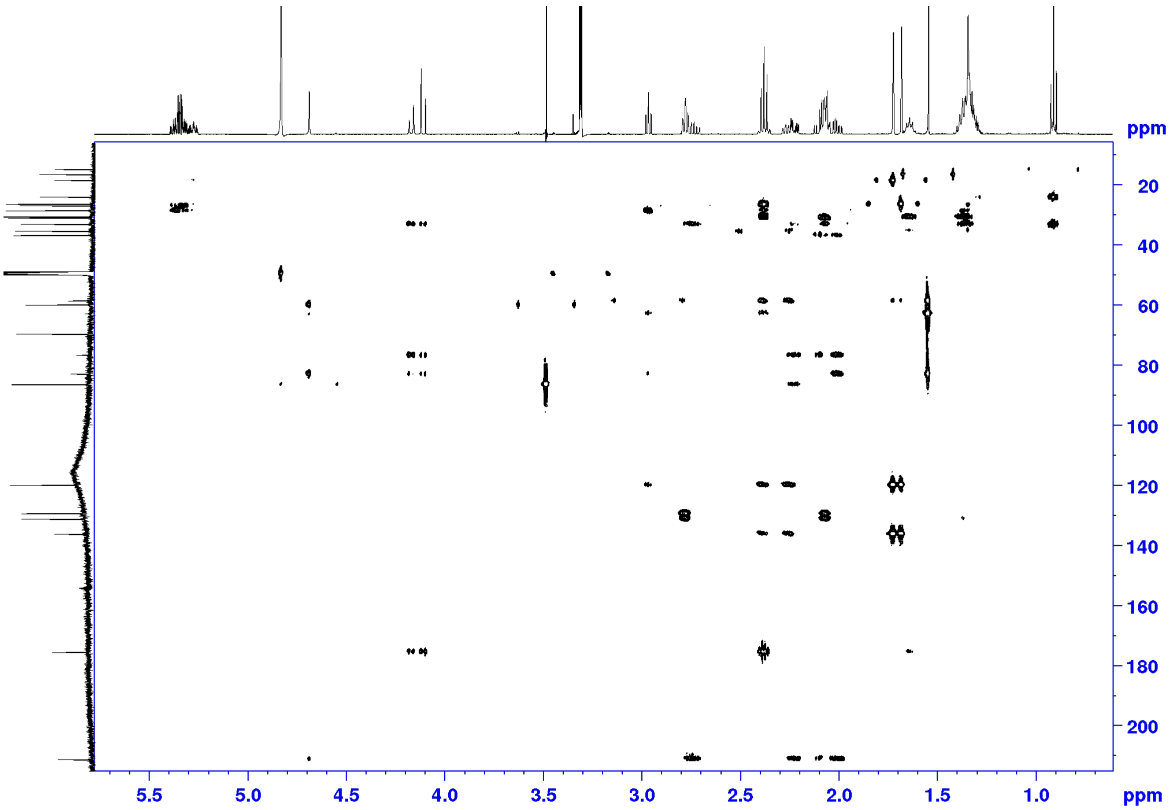


*Figure S8.* *NOESY NMR (500 MHz, $CD_3OD$) spectrum of ovalicin linoleate (**2**)*

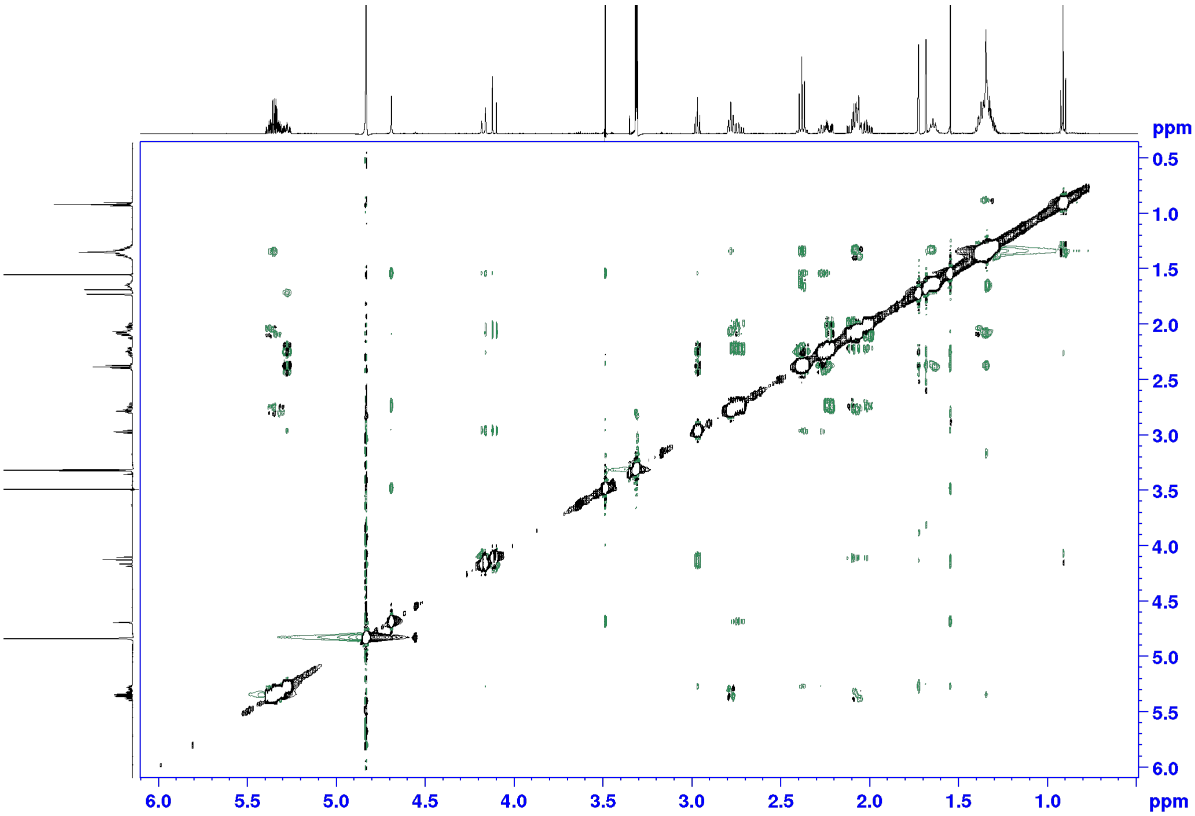

*Figure S9.*: *ESI-HRMS spectra of* ***2***

**Elemental Composition Report** Page 1 of 1

**Single Mass Analysis**
Tolerance = 5.0 PPM / DBE: min = -1.5, max = 100.0
Element prediction: Off
Number of isotope peaks used for i-FIT = 9

Monoisotopic Mass, Even Electron Ions
996 formula(e) evaluated with 4 results within limits (all results (up to 1000) for each mass)
Elements Used:
C: 1-120 H: 1-150 N: 0-10 O: 0-15 Na: 1-1
STIEN Isorres31-1 831 (3.859) Cm (818:870)
1: TOF MS ES+

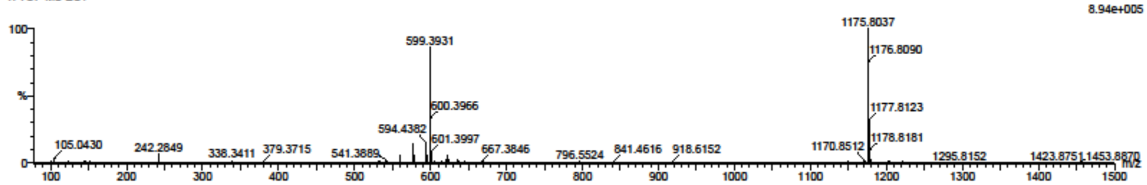


| | | | | | | | |
|---|---|---|---|---|---|---|---|
| Minimum: | | | | -1.5 | | | |
| Maximum: | | 5.0 | 5.0 | 100.0 | | | |
| Mass | Calc. Mass | mDa | PPM | DBE | i-FIT | i-FIT (Norm) | Formula |
| 599.3931 | 599.3924 | 0.7 | 1.2 | 6.5 | 2226.0 | 0.1 | C34 H56 O7 Na |
| | 599.3937 | -0.6 | -1.0 | 11.5 | 2229.0 | 3.0 | C35 H52 N4 O3 Na |
| | 599.3910 | 2.1 | 3.5 | 12.5 | 2231.0 | 5.0 | C31 H48 N10 O Na |
| | 599.3956 | -2.5 | -4.2 | -1.5 | 2233.7 | 7.7 | C23 H56 N6 O10 Na |

*Table S3. Full NMR data ovalicin oleate (**3**) recorded in $CD_3OD$*

| Position | $\delta_C$, Type | $\delta_H$ (*J* in Hz) | COSY | HMBC |
|---|---|---|---|---|
| 1 | 211.1, C | | | |
| 2a | 36.5, $CH_2$ | 2.74, bsex (7.3) | H2b, H3 | C1, C3 |
| 2b | | 2.22, m | H2a, H3 | C1, C3, C4, C7 |
| 3a | 32.7, $CH_2$ | 2.09, m | H2a, H2b, H3b | C1, C2, C4 |
| 3b | | 2.01, m | H2a, H2b, H3a | C1, C2, C4, C5 |
| 4 | 76.5, C | | | |
| 5 | 82.7, C | | | |
| 6 | 62.7*, C | | | |
| 7 | 86.2, CH | 4.69, s | | C1, C5, OMe |
| 8 | 58.3, CH | 2.97, t (6.5) | H9a, H9b | C5, C6, C9, C10 |
| 9a | 28.4, $CH_2$ | 2.37, m | H8, H9b, H10, H12 (LR) | C8, C10, C11 |
| 9b | | 2.27, m | H8, H9a, H10, H12 (LR) | C8, C10, C11 |
| 10 | 119.8, CH | 5.27, m | H9a, H9b, H12 (LR), H13 (LR) | C12 (W), C13 (W) |
| 11 | 136.1, C | | | |
| 12 | 26.1, $CH_3$ | 1.73, bd (1.1) | H9a, H9b, H10 | C10, C11, C13 |
| 13 | 18.2, $CH_3$ | 1.68, bd (0.8) | H10 | C10, C11,C12 |
| 14a | 69.4, $CH_2$ | 4.17, d (11.1) | H14b | C3, C4, C5, C1' |
| | | 4.11, d (11.1) | H14a | C3, C4, C5, C1' |
| 15 | 16.3, $CH_3$ | 1.55, s | | C5, C6, C8 |
| OMe | 59.7, $CH_3$ | 3.49, s | | C7 |
| 1' | 175.4, C | | | |
| 2' | 35.1, CH2 | 2.38, m | H3' | C1', C3', C4' |
| 3' | 26.1, CH2 | 1.64, m | H2', H4' | C1' |
| 4' – 7' | 30.2 – 31.0, CH2 | 1.26 – 1.40, m | | |
| 8' | 28.3 | 2.03, m | H7', H9' | C9', C10' |
| 9' | 130.9 | 5.35, m | | C8', C11' |
| 10' | 131.1 | 5.35, m | | C8', C11' |
| 11' | 28.3 | 2.03, m | H10', H12' | C9', C10' |
| 12' – 15' | 30.2 – 31.0, CH2 | 1.26 – 1.40, m | | |
| 16' | 33.2 | 1.30, m | | |
| 17' | 23.9 | 1.29, m | H18' | |
| 18' | 14.6 | 0.90, t (7.0) | H17' | C17', C16' |

LR: long range
W: Weak
*: deduced from HMBC correlations

*Figure S10.* ¹H NMR (500 MHz, $CD_3OD$) spectrum of ovalicin oleate (**3**)

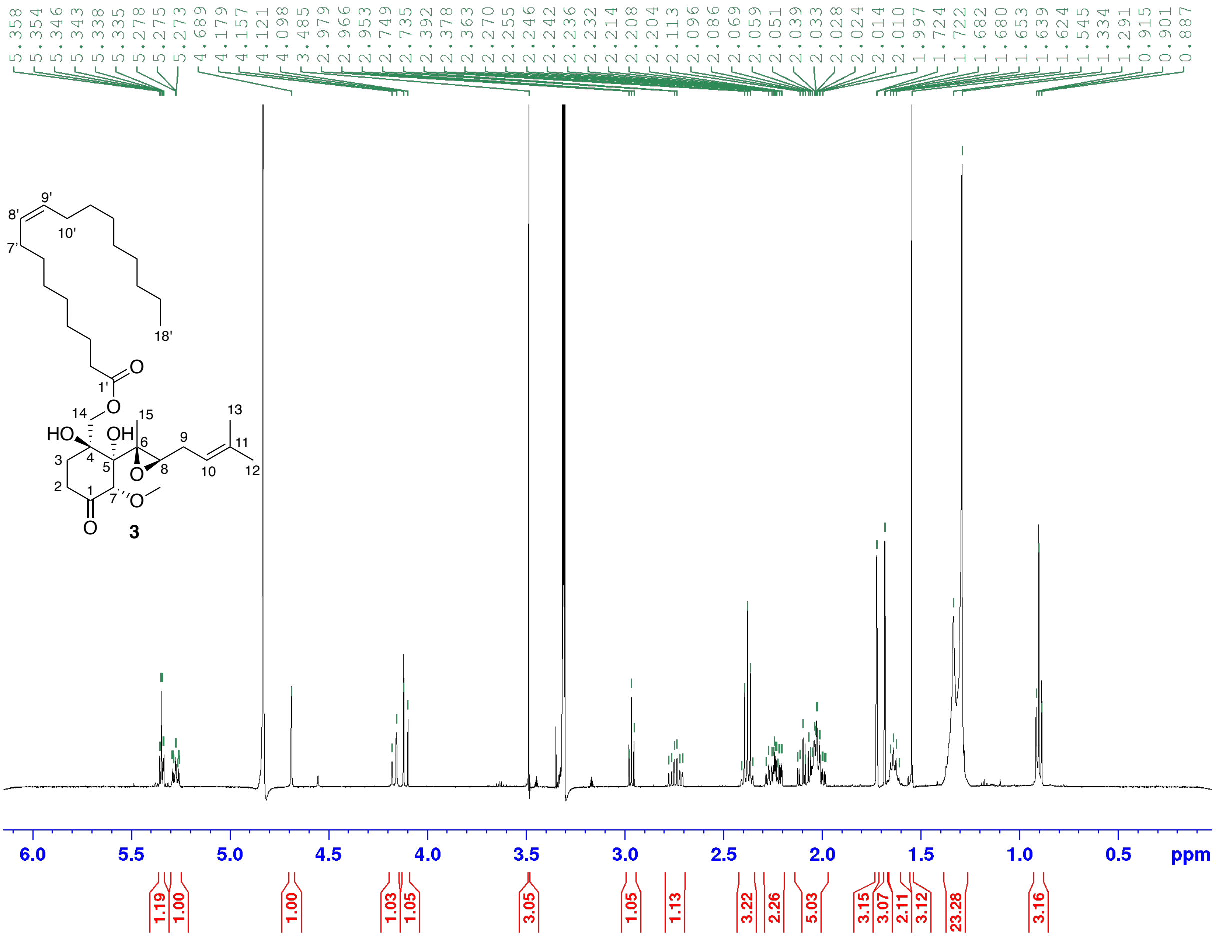

*Figure S11.* $^{13}$*C NMR (125 MHz, CD$_3$OD) spectrum of ovalicin oleate (**3**)*

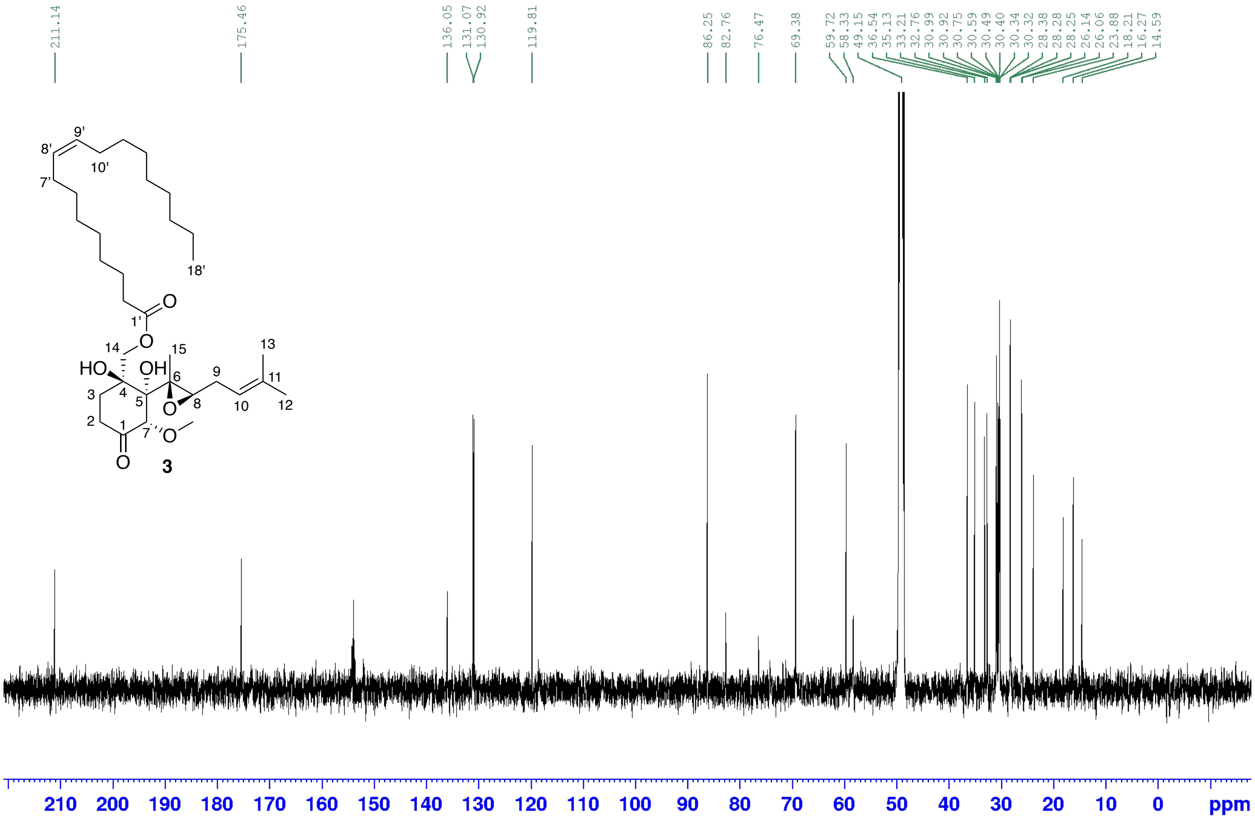


*Figure S12. COSY NMR (500 MHz, CD$_3$OD) spectrum of ovalicin oleate (**3**)*

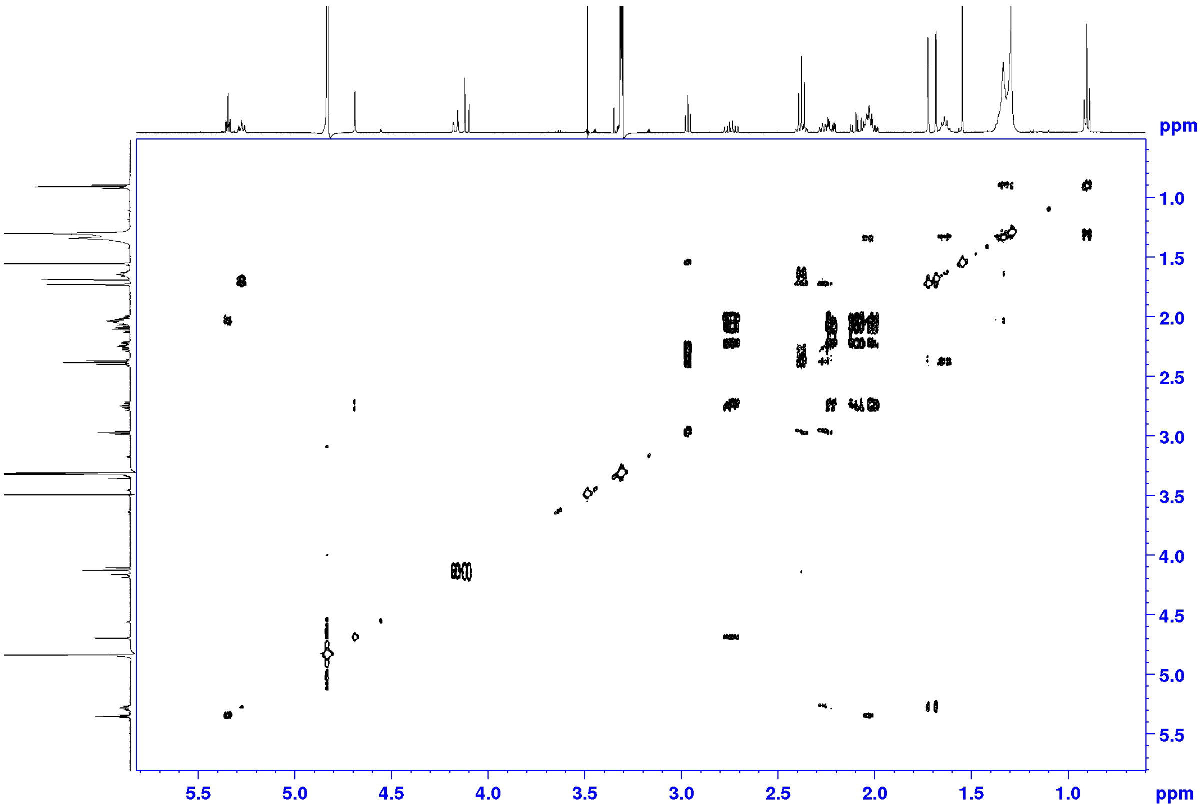

*Figure S13.* *HSQC NMR (500 MHz, $CD_3OD$) spectrum of ovalicin oleate (**3**)*

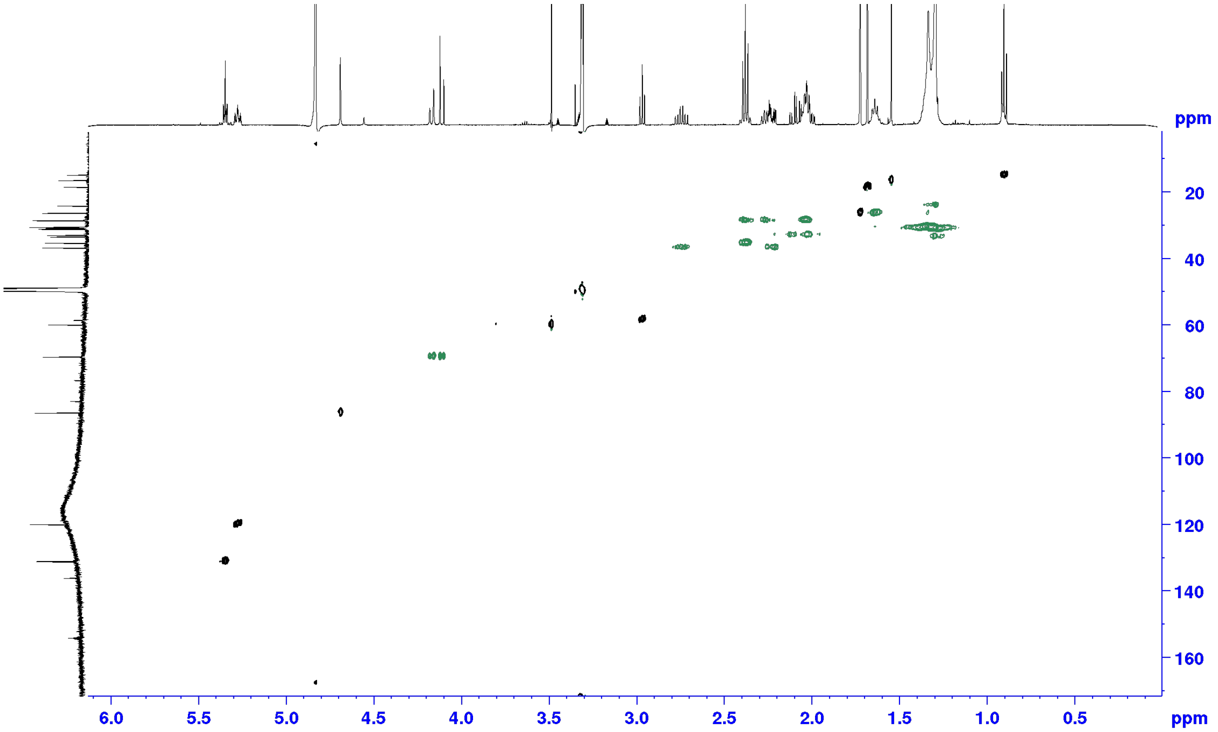


*Figure S14.* *HMBC NMR (500 MHz, $CD_3OD$) spectrum of ovalicin oleate (**3**)*

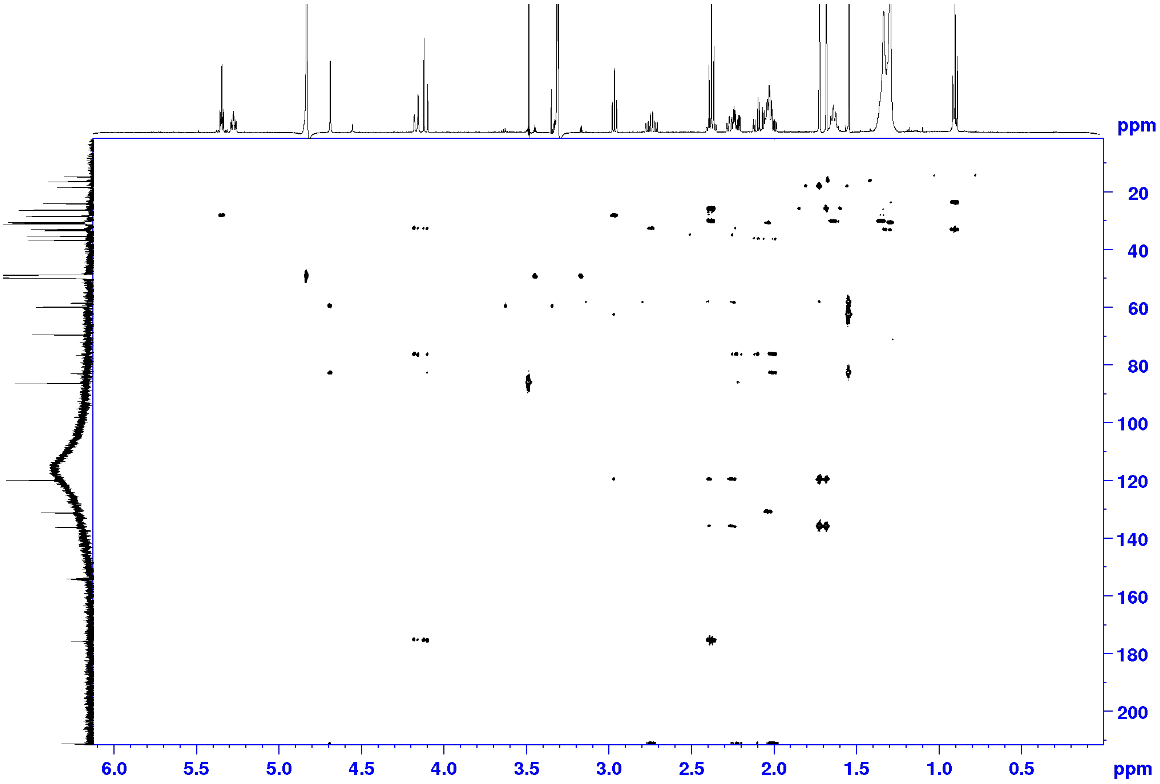

*Figure S15*: *ESI-HRMS spectra of* ***3***

**Single Mass Analysis**
Tolerance = 5.0 PPM / DBE: min = -1.5, max = 100.0
Element prediction: Off
Number of isotope peaks used for i-FIT = 9
Monoisotopic Mass, Even Electron Ions
502 formula(e) evaluated with 2 results within limits (all results (up to 1000) for each mass)
Elements Used:

| Mass | Calc. Mass | mDa | PPM | DBE | Formula | i-FIT | i-FIT (Norm) | C | H | N | O | Na |
|---|---|---|---|---|---|---|---|---|---|---|---|---|
| 601.4091 | 601.4080 | 1.1 | 1.8 | 5.5 | C34 H58 O7 Na | 212.8 | 0.4 | 34 | 58 | | 7 | 1 |
| | 601.4094 | -0.3 | -0.5 | 10.5 | C35 H54 N4 O3 Na | 213.7 | 1.2 | 35 | 54 | 4 | 3 | 1 |

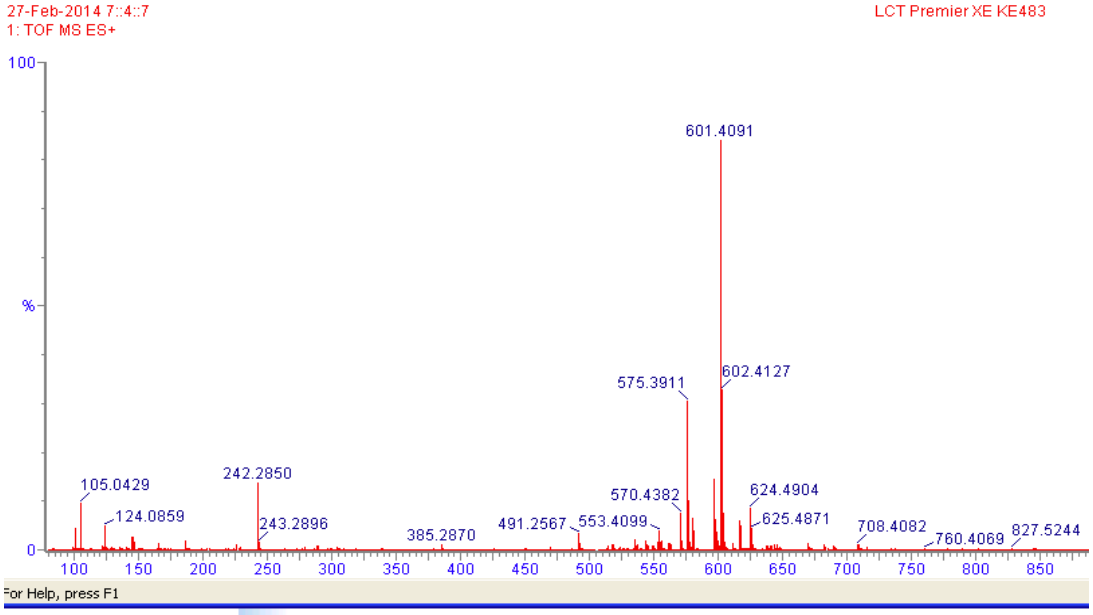

*Table S4. Full NMR data ovalicin stearate (**4**) recorded in $CD_3OD$*

| Position | $\delta_C$, Type | $\delta_H$ (*J* in Hz) | COSY | HMBC |
|---|---|---|---|---|
| 1 | 211.2, C | | | |
| 2a | 36.5, $CH_2$ | 2.74, td (13.7; 7.6) | H2b, H3 | C1, C3 |
| 2b | | 2.23, m | H2a, H3 | C1, C3, C4, C7 |
| 3a | 32.8, $CH_2$ | 2.09, td (13.6; 7.1) | H2a, H2b, H3b | C1, C2, C4 |
| 3b | | 2.01, ddd (13.6; 7.1; 1.9) | H2a, H2b, H3a | C1, C2, C4, C5 |
| 4 | 76.5*, C | | | |
| 5 | 82.8, C | | | |
| 6 | 62.7*, C | | | |
| 7 | 86.2, CH | 4.69, s | | C5, OMe |
| 8 | 58.4, CH | 2.97, t (6.5) | H9a, H9b | C6, C9 |
| 9a | 28.4, $CH_2$ | 2.38, m | H8, H9b, H10, H12 (LR) | C10, C11 |
| 9b | | 2.25, m | H8, H9a, H10, H12 (LR) | C10, C11 |
| 10 | 119.8, CH | 5.27, m | H9a, H9b, H12 (LR), H13 (LR) | |
| 11 | 136.1, C | | | |
| 12 | 26.0, $CH_3$ | 1.73, bs | H9a (LR), H9b (LR), H10 (LR) | C10, C11, C13 |
| 13 | 18.2, $CH_3$ | 1.68, bs | H10 | C10, C11, C12 |
| 14a | 69.4, $CH_2$ | 4.17, d (11.1) | H14b | C3, C4, C1' |
| 14b | | 4.11, d (11.1) | H14a | C3, C1' |
| 15 | 16.3, $CH_3$ | 1.54, s | | C5, C6, C8 |
| OMe | 59.7, $CH_3$ | 3.48, s | | C7 |
| 1' | 175.5, C | | | |
| 2' | 35.1, $CH_2$ | 2.38, m | H3' | C1', C3', C4' |
| 3' | 26.4, $CH_2$ | 1.64, m | H2', H4' | |
| 4' – 15' | 30.3 – 31.0, $CH_2$ | 1.25 – 1.37, m | | |
| 16' | 33.2, $CH_2$ | 1.29, m | | |
| 17' | 23.9, $CH_2$ | 1.29, m | H18' | |
| 18' | 14.6, $CH_3$ | 0.90, t (7.0) | H17' | C17', C16' |

*Figure S16.* $^{1}$H NMR (500 MHz, $CD_3OD$) spectrum of ovalicin stearate (**4**)

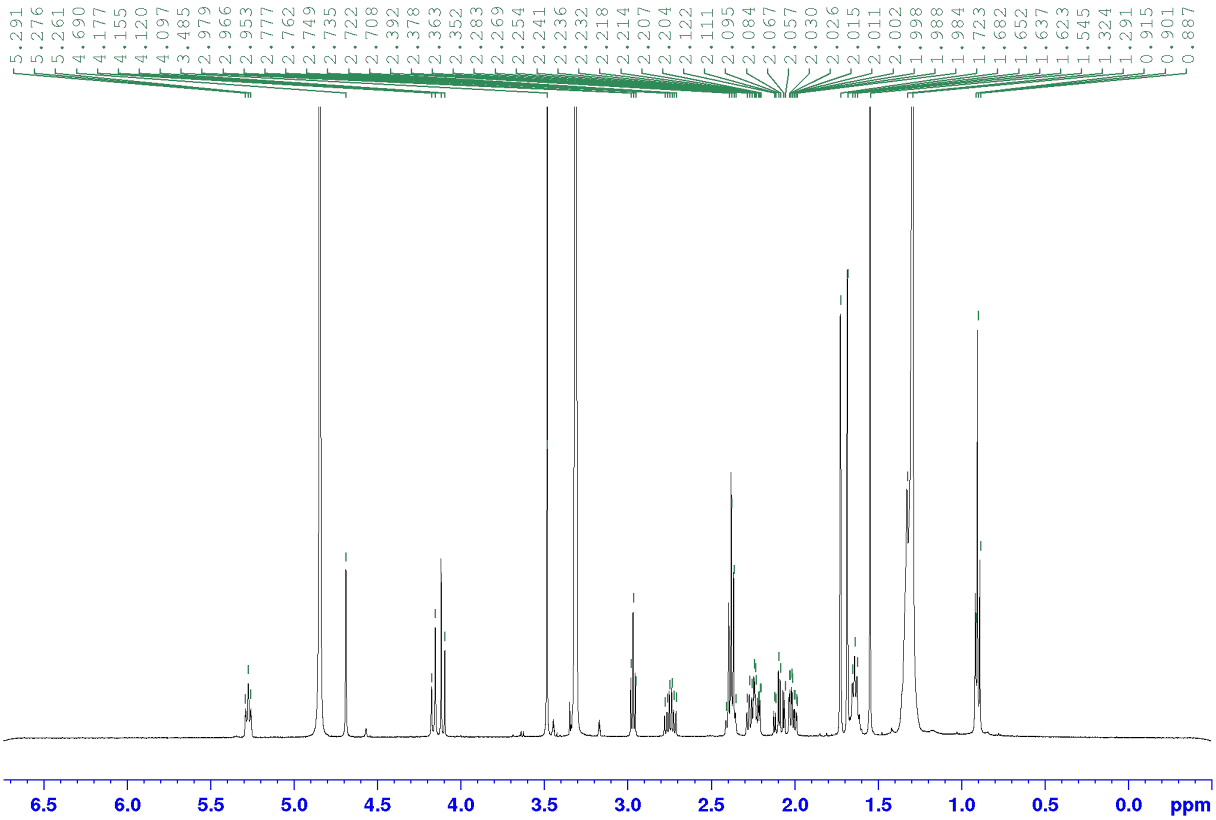


*Figure S17.* $^{13}$C NMR (125 MHz, $CD_3OD$) spectrum of ovalicin stearate (**4**)

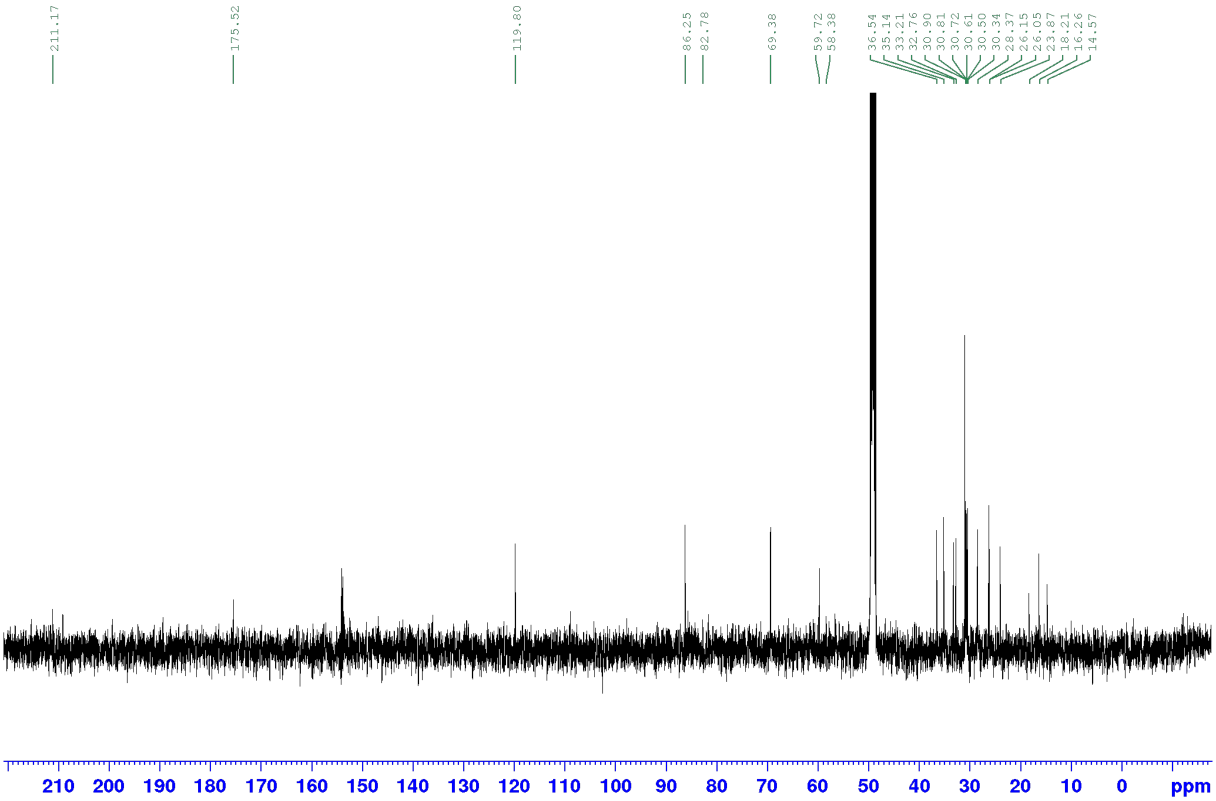

*Figure S18.* COSY (500 MHz, $CD_3OD$) spectrum of ovalicin stearate (**4**)

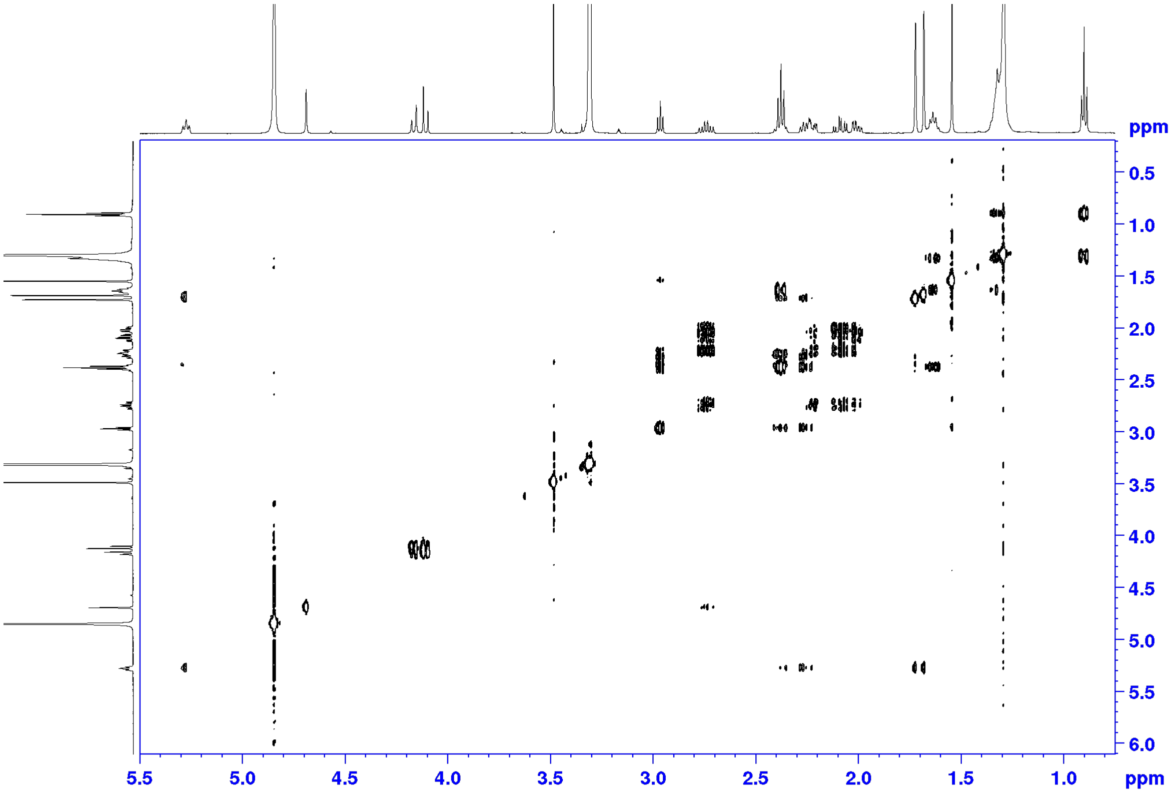


*Figure S19.* HMQC (500 MHz, $CD_3OD$) spectrum of ovalicin stearate (**4**)

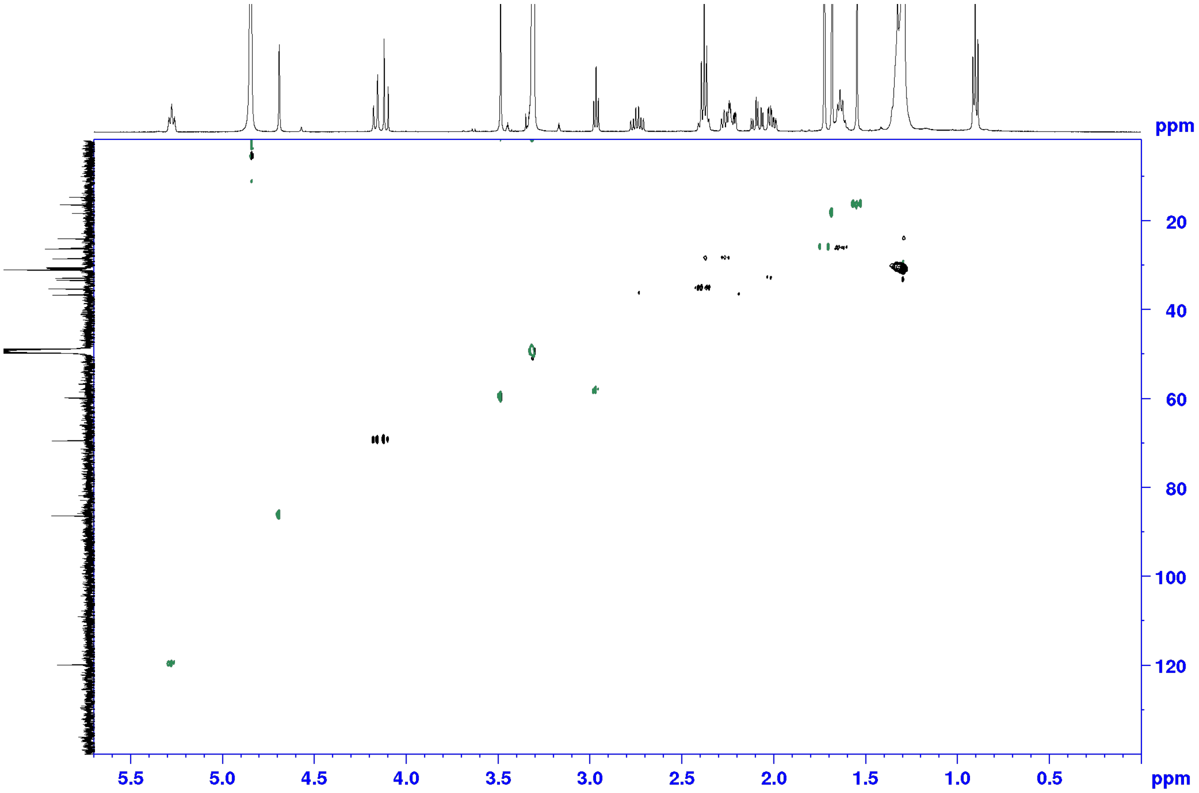

*Figure S20*. *HMBC (500 MHz, $CD_3OD$) spectrum of ovalicin stearate (**4**)*

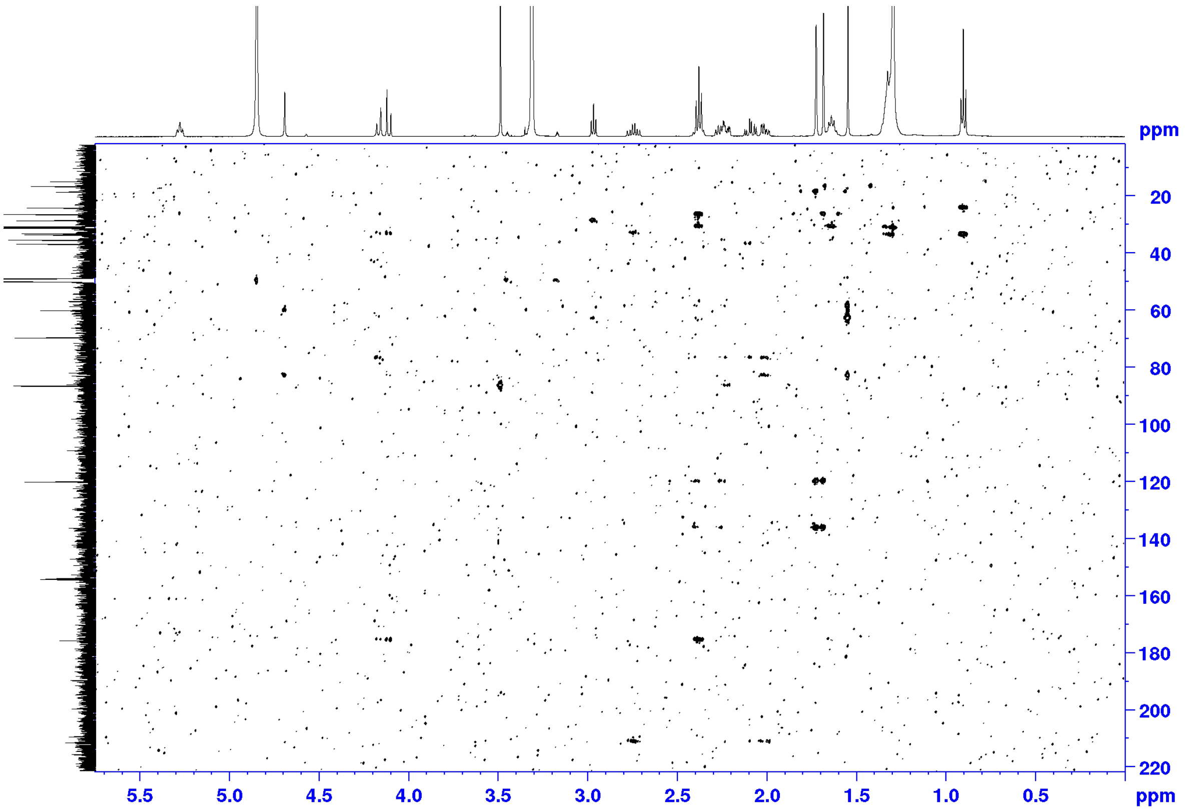


*Figure S21*: *ESI-HRMS spectra of **4***

**Single Mass Analysis**
Tolerance = 5.0 PPM / DBE: min = -1.5, max = 100.0
Element prediction: Off
Number of isotope peaks used for i-FIT = 9
Monoisotopic Mass, Even Electron Ions
503 formula(e) evaluated with 3 results within limits (all results (up to 1000) for each mass)
Elements Used:

| Mass | Calc. Mass | mDa | PPM | DBE | Formula | i-FIT | i-FIT (Norm) | C | H | N | O | Na |
|---|---|---|---|---|---|---|---|---|---|---|---|---|
| 603.4235 | 603.4237 | -0.2 | -0.3 | 4.5 | C34 H60 O7 Na | 386.0 | 0.8 | 34 | 60 | | 7 | 1 |
| | 603.4250 | -1.5 | -2.5 | 9.5 | C35 H56 N4 O3 Na | 386.4 | 1.2 | 35 | 56 | 4 | 3 | 1 |
| | 603.4210 | 2.5 | 4.1 | 5.5 | C30 H56 N6 O5 Na | 386.6 | 1.4 | 30 | 56 | 6 | 5 | 1 |

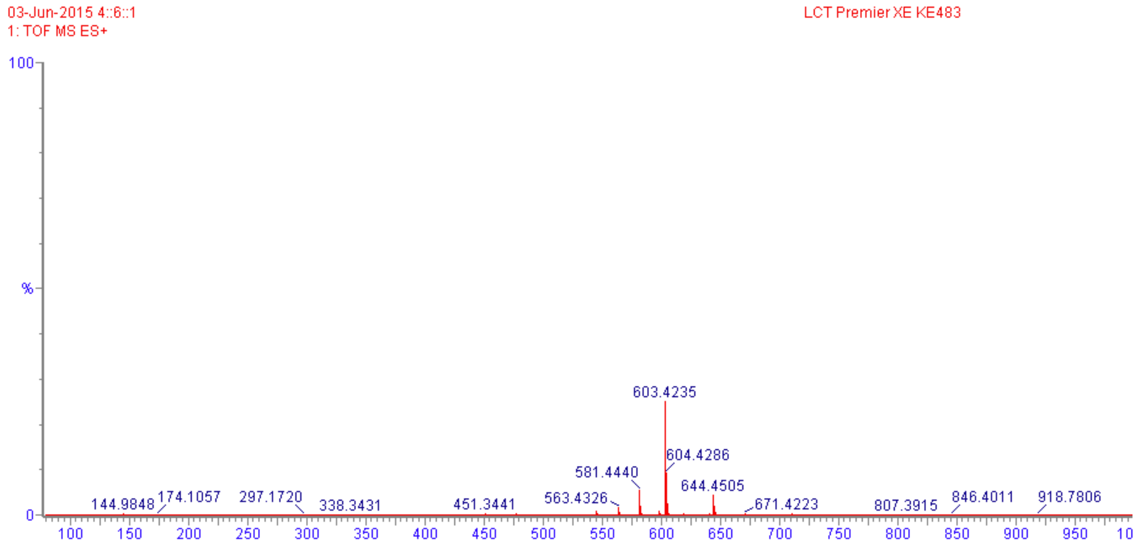

*Figure S22.* *$^{1}H$ NMR (800 MHz, $CD_3OD$ with water suppression) spectrum of ovalicin palmitoleate (**5**)*

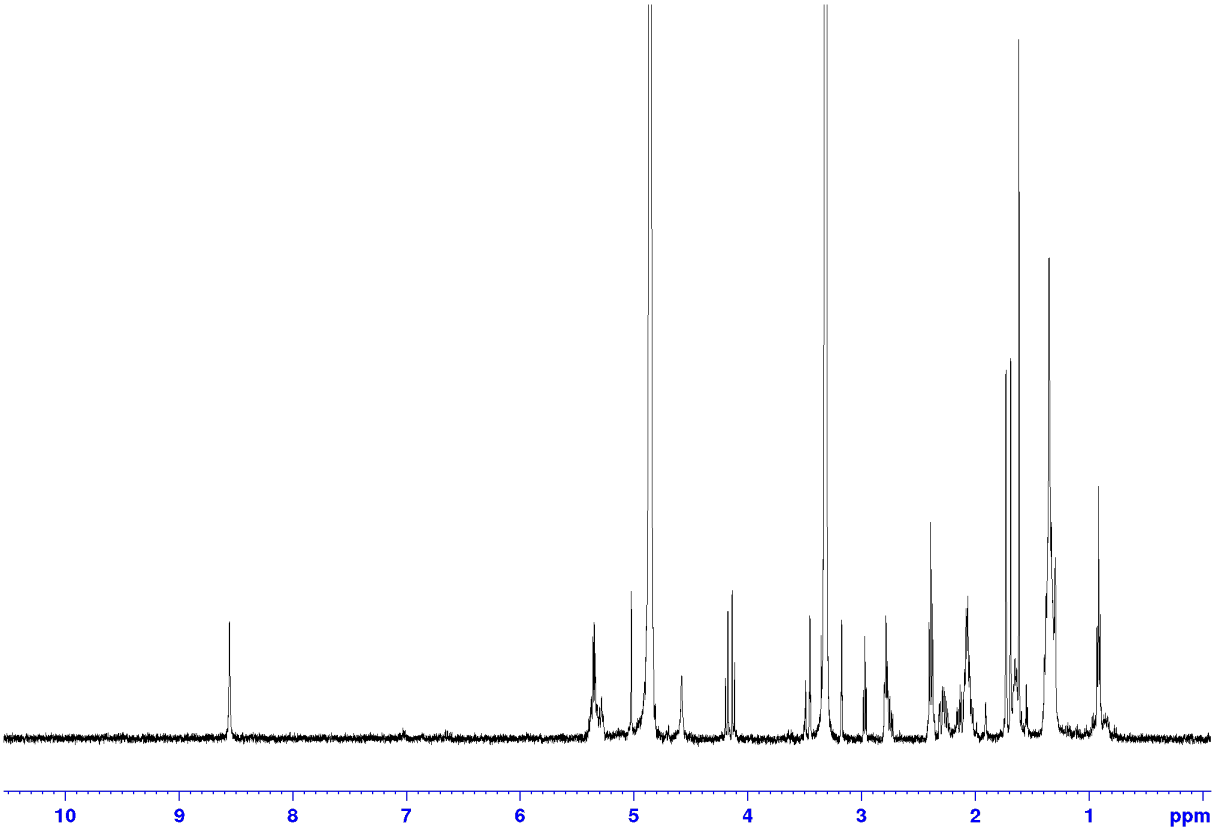


*Figure S23.* *ESI-HRMS spectra of **5***

**Single Mass Analysis**
Tolerance = 5.0 PPM / DBE: min = -1.5, max = 100.0
Element prediction: Off
Number of isotope peaks used for i-FIT = 9

Monoisotopic Mass, Even Electron Ions
94 formula(e) evaluated with 1 results within limits (all results (up to 1000) for each mass)
Elements Used:
C: 1-100 H: 0-100 O: 0-15 Na: 1-1

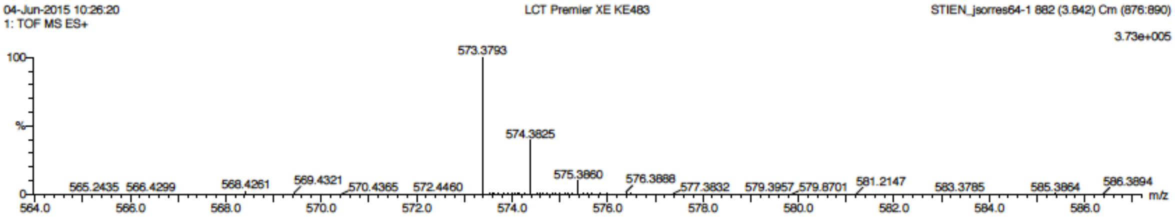


| | | | | | | | |
|---|---|---|---|---|---|---|---|
| Minimum: | | | | -1.5 | | | |
| Maximum: | | 10.0 | 5.0 | 100.0 | | | |
| Mass | Calc. Mass | mDa | PPM | DBE | i-FIT | i-FIT (Norm) | Formula |
| 573.3793 | 573.3767 | 2.6 | 4.5 | 5.5 | 1365.8 | 0.0 | C32 H54 O7 Na |

*Table S5. Full NMR data (500 MHz, $CD_3OD$) of demethylovalicin linoleate (**6**)*

| Position | $\delta_C$, Type | $\delta_H$ (*J* in Hz) | COSY | HMBC |
|---|---|---|---|---|
| 1 | 211.6, C | | | |
| 2a | 36.5, $CH_2$ | 2.76, m | H2b, H3 | C1, C3, C4, C7 |
| 2b | | 2.29, m | H2a, H3 | C1, C3 |
| 3a | 32.8, $CH_2$ | 2.12, m | H2a, H2b, H3b | |
| 3b | | 2.04, m | H2a, H2b, H3a | C1, C4 |
| 4 | 76.3*, C | | | |
| 5 | 81.8*, C | | | |
| 6 | 62.3*, C | | | |
| 7 | 77.5, CH | 5.02, s | | C1, C5 |
| 8 | 57.9*, CH | 2.96, t (6.8) | H9a, H9b | C9 |
| 9a | 28.4, $CH_2$ | 2.38, m | H8, H9b, H10, H12 (LR), H13 (LR) | C6, C10, C11 |
| 9b | | 2.26, m | H8, H9a, H10, H12 (LR), H13 (LR) | C8, C10, C11 |
| 10 | 119.9, CH | 5.28, m | H9a, H9b, H12 (LR), H13 (LR) | |
| 11 | 136.0, C | | | |
| 12 | 26.1, $CH_3$ | 1.72, bs | H9a (LR), H9b (LR), H10 (LR) | C10, C11, C13 |
| 13 | 18.2, $CH_3$ | 1.68, bs | H9a (LR, W), H9b (LR, W), H10 (LR) | C10, C11, C12 |
| 14a | 69.5, $CH_2$ | 4.18, d (11.1) | H14b | C3, C4, C1' |
| | | 4.12, d (11.1) | H14a | C3, C1' |
| 15 | 16.3, $CH_3$ | 1.61, s | | C5, C6, C8 |
| 1' | 175.2, C | | | |
| 2' | 35.1, $CH_2$ | 2.38, m | H3' | C1', C3' |
| 3' | 26.2, $CH_2$ | 1.64, m | H2' | C2' |
| 4' – 6' | 30.2 – 30.8, $CH_2$ | 1.27 – 1.40, m | | |
| 7' | 30.4, $CH_2$ | 1.37, m | | |
| 8' | 28.4, $CH_2$ | 2.07, m | H7', H9' | C7', C9', C10' |
| 9' | 131.0, CH | 5.36, m | H8', H10' | |
| 10' | 129.3, CH | 5.33, m | H9', H11' | |
| 11' | 26.7, $CH_2$ | 2.77, m | H10', H12' | C9', C10', C12', C13' |
| 12' | 129.2, CH | 5.33, m | H11', H13' | |
| 13' | 131.1, CH | 5.36, m | H12', H14' | |
| 14' | 28.4, $CH_2$ | 2.07, m | H13', H15' | |
| 15' | 30.4, $CH_2$ | 1.37, m | | |
| 16' | 32.8, $CH_2$ | 1.31, m | | |
| 17' | 23.8, $CH_2$ | 1.33, m | H18' | |
| 18' | 14.6 | 0.91, t (7.2) | H17' | C17', C16' |

*Figure S24.* $^{1}$H NMR (800 MHz, $CD_3OD$ with water suppression) spectrum of demethylovalicin linoleate (**6**)

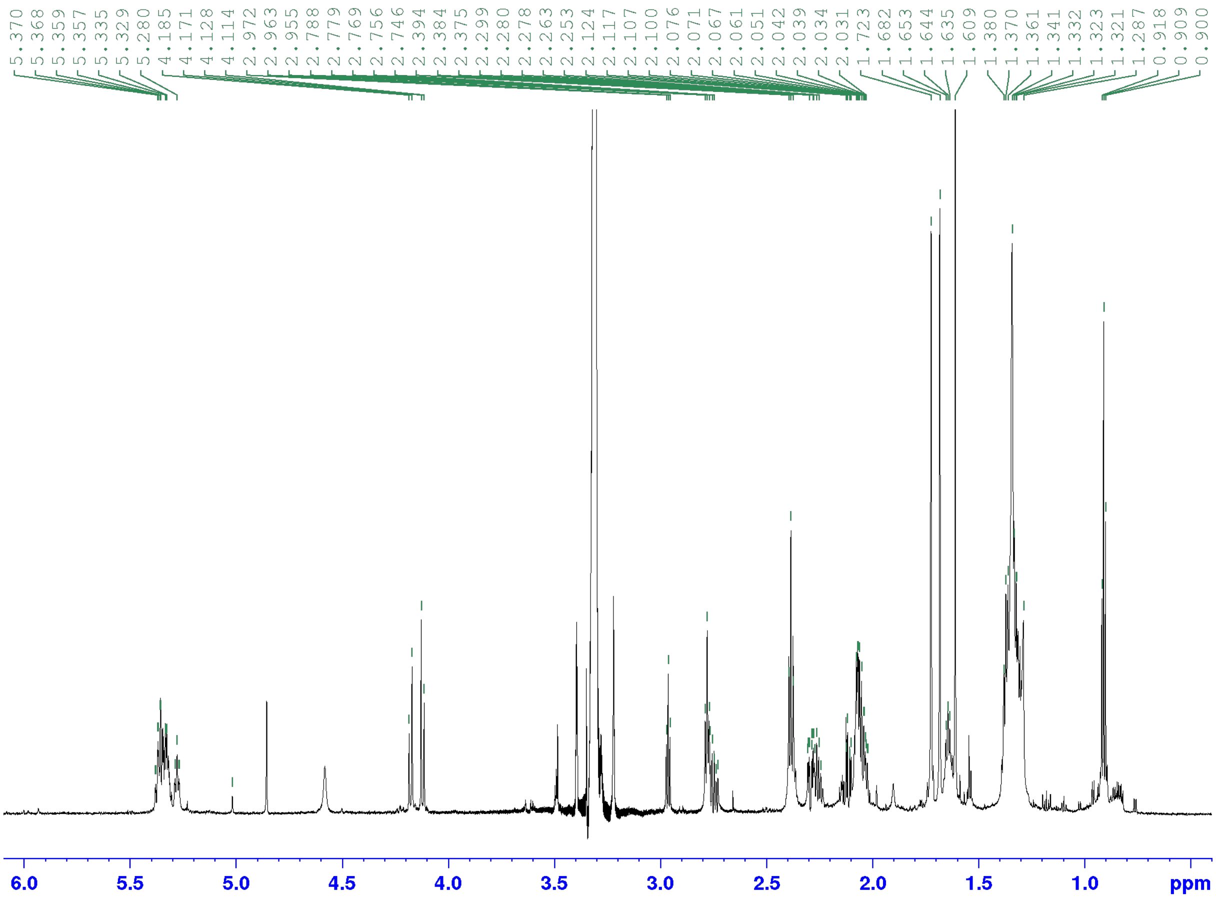

*Figure S25.* $^{13}$C NMR (200 MHz, $CD_3OD$) spectrum of demethylovalicin linoleate (**6**)

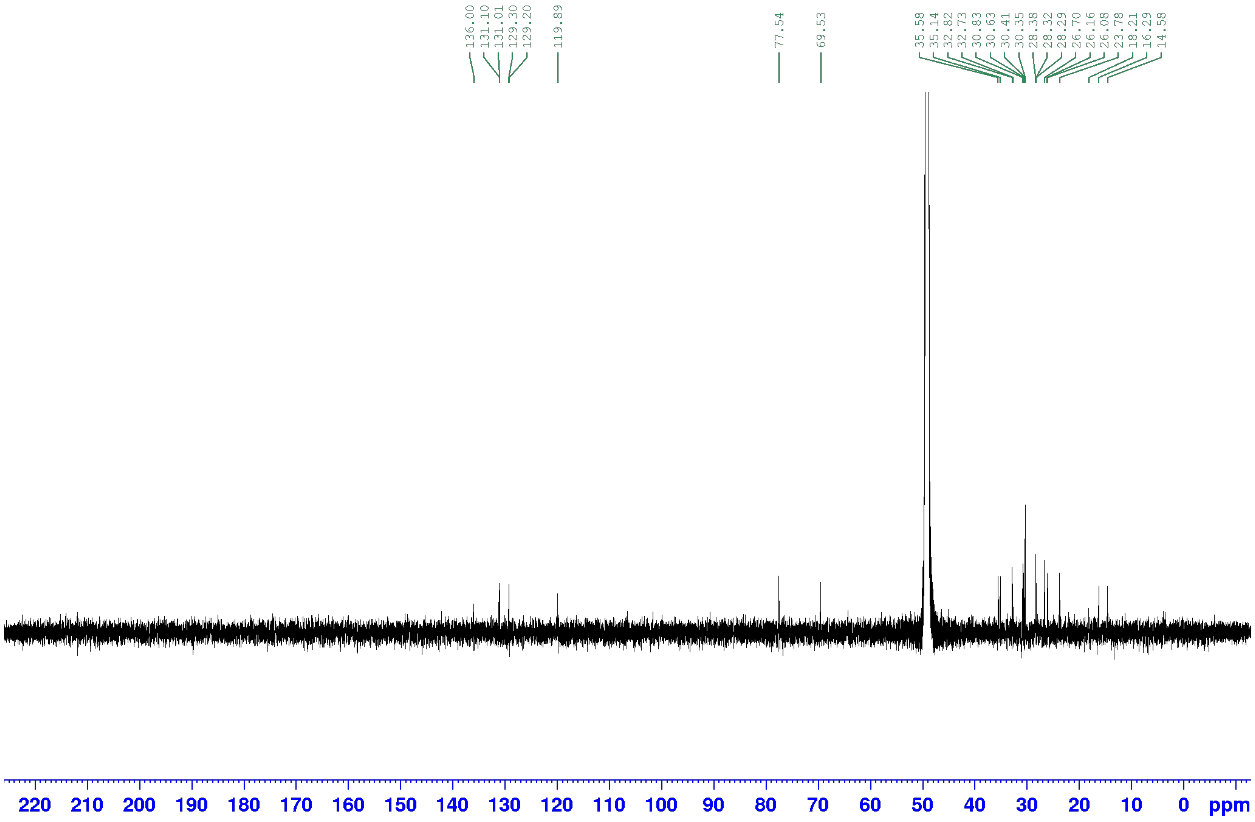

*Figure S26. COSY (800 MHz, $CD_3OD$) spectrum of demethylovalicin linoleate (**6**)*

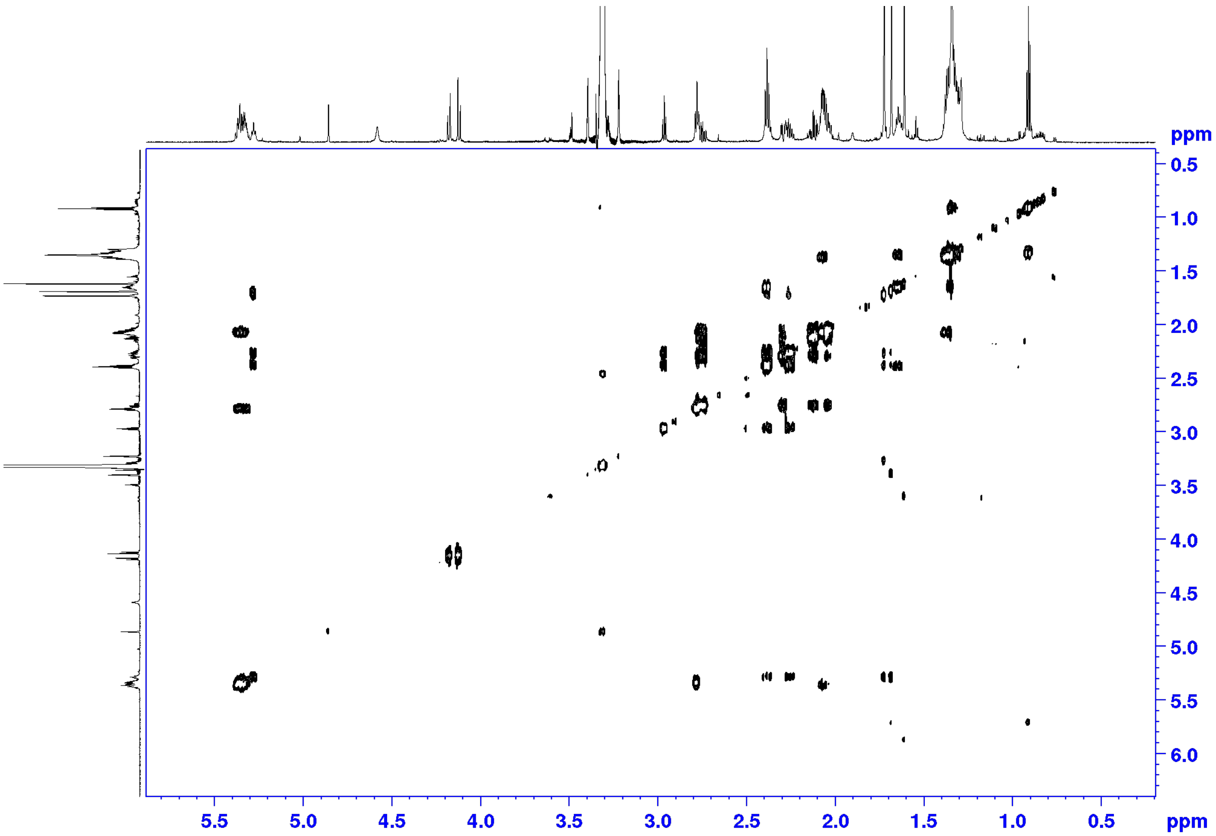


*Figure S27. HSQC (800 MHz, $CD_3OD$) spectrum of demethylovalicin linoleate (**6**)*

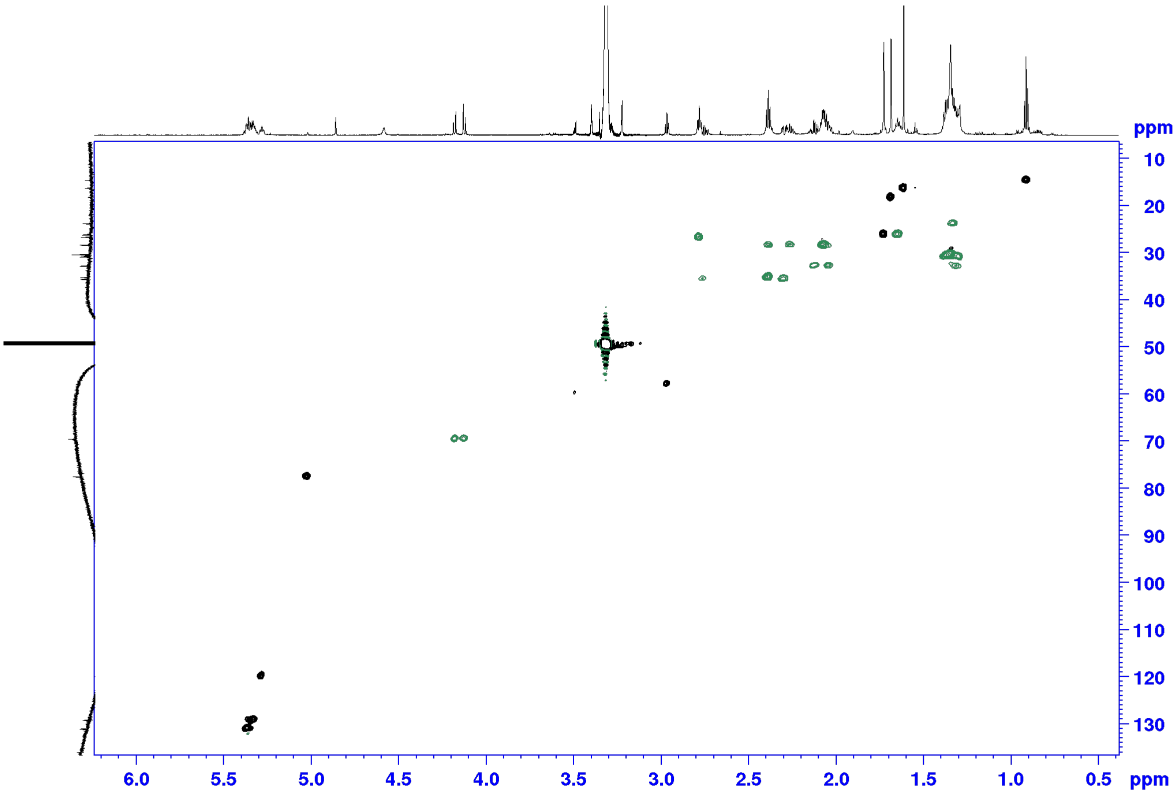

*Figure S28. HMBC (800 MHz, $CD_3OD$) spectrum of demethylovalicin linoleate 6)*

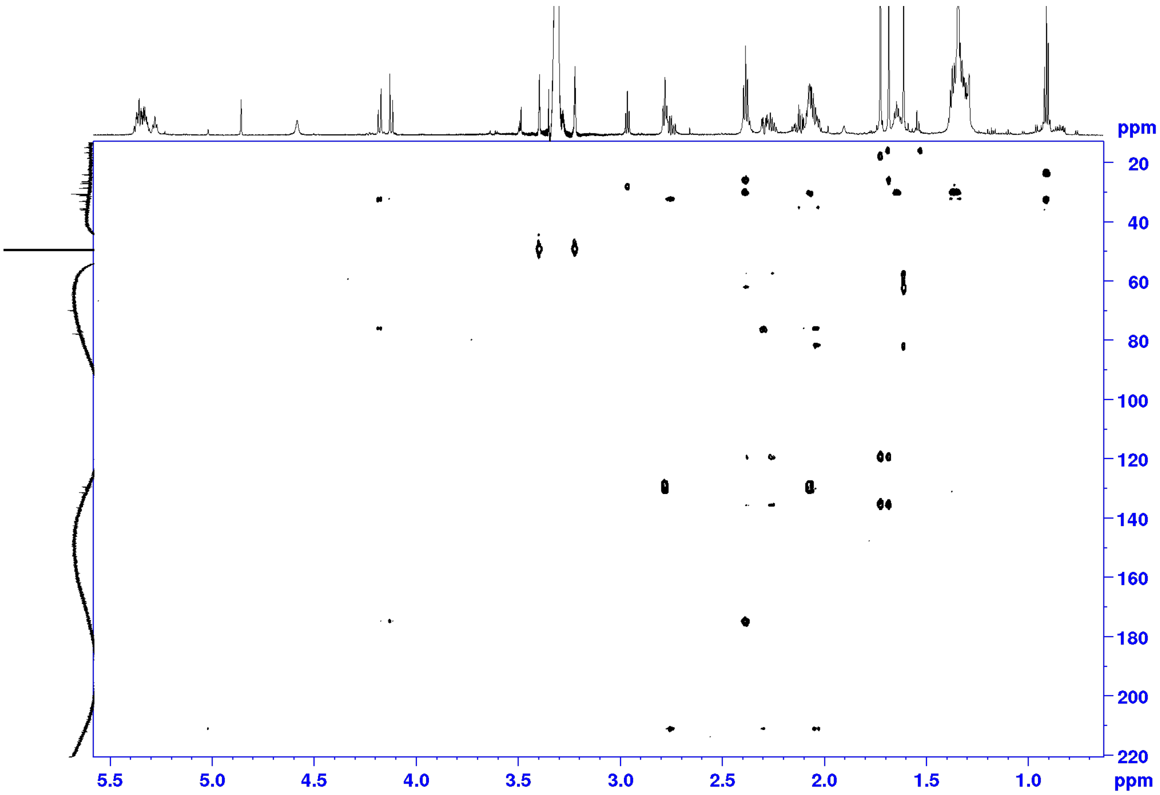


*Figure S29: ESI-HRMS spectra of **6***

**Elemental Composition Report** **Page 1 of 1**

**Single Mass Analysis**

Tolerance = 5.0 PPM / DBE: min = -1.5, max = 100.0

Element prediction: Off

Number of isotope peaks used for i-FIT = 9

Monoisotopic Mass, Even Electron Ions

970 formula(e) evaluated with 4 results within limits (all results (up to 1000) for each mass)

Elements Used:

C: 1-120 H: 1-150 N: 0-10 O: 0-15 Na: 1-1

STIEN |sorres61-1 884 (3.850) Cm (866:899)

1: TOF MS ES+

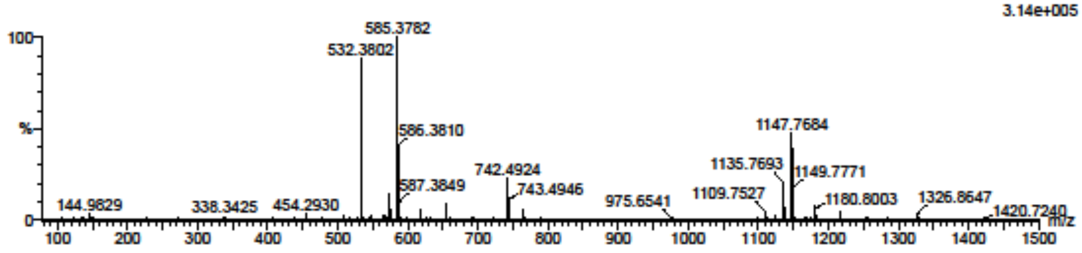


| | | | | | | | |
|---|---|---|---|---|---|---|---|
| Minimum: | | | | -1.5 | | | |
| Maximum: | | 5.0 | 5.0 | 100.0 | | | |
| Mass | Calc. Mass | mDa | PPM | DBE | i-FIT | i-FIT (Norm) | Formula |
| 585.3782 | 585.3767 | 1.5 | 2.6 | 6.5 | 1785.3 | 0.7 | C33 H54 O7 Na |
| | 585.3781 | 0.1 | 0.2 | 11.5 | 1785.5 | 0.9 | C34 H50 N4 O3 Na |
| | 585.3754 | 2.8 | 4.8 | 12.5 | 1786.9 | 2.3 | C30 H46 N10 O Na |
| | 585.3799 | -1.7 | -2.9 | -1.5 | 1790.0 | 5.4 | C22 H54 N6 O10 Na |

*Figure S30. HRMS analyses of **2***

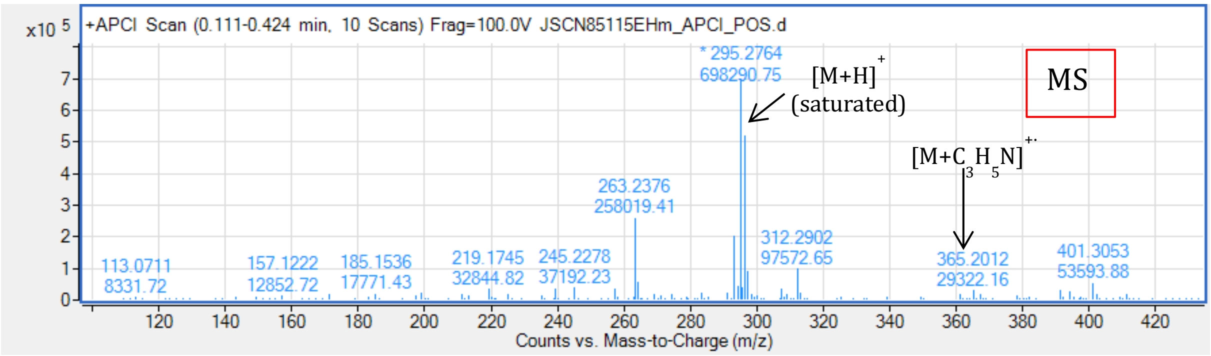


| Best | Formula | Score | Mass | ID Source | Diff (ppm) | Mass (MFG) | Diff (mDa) | DBE |
|---|---|---|---|---|---|---|---|---|
| ⦿ | C22 H39 N O2 | 94.38 | 349.2988 | MFG | -2.01 | 349.2981 | -0.7 | 4 |

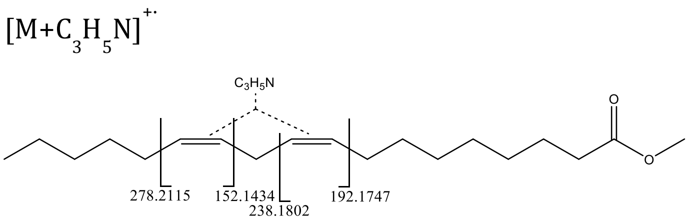


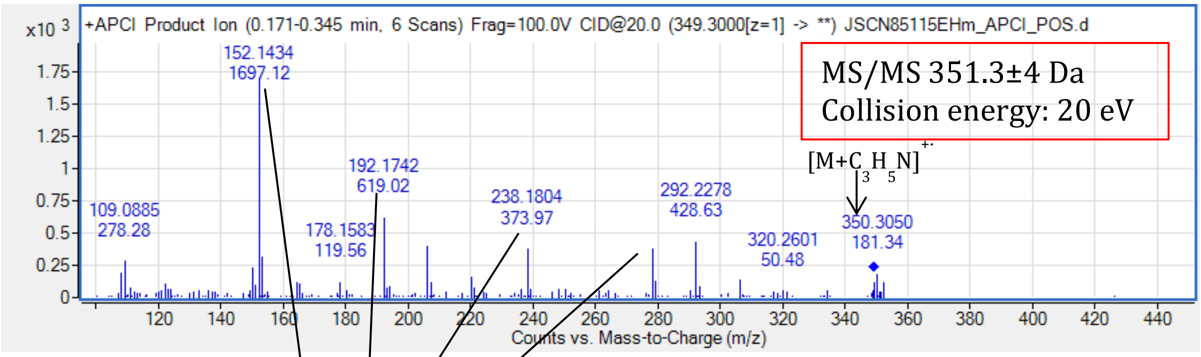


| Best | Formula | Score | Mass | ID Source | Diff (ppm) | Mass (MFG) | Diff (mDa) | DBE |
|---|---|---|---|---|---|---|---|---|
| ○ | C10 H18 N | 71.49 | 152.1442 | MFG | -1.72 | 152.1439 | -0.26 | 2.5 |
| ⦿ | C13 H22 N | 83.62 | 192.1751 | MFG | 0.73 | 192.1752 | 0.14 | 3.5 |
| ○ | C14 H24 N O2 | 83.08 | 238.1809 | MFG | -0.88 | 238.1807 | -0.21 | 3.5 |
| ○ | C17 H28 N O2 | 70.02 | 278.2126 | MFG | -1.98 | 278.212 | -0.55 | 4.5 |

*Figure S31. HRMS analyses of **2b***

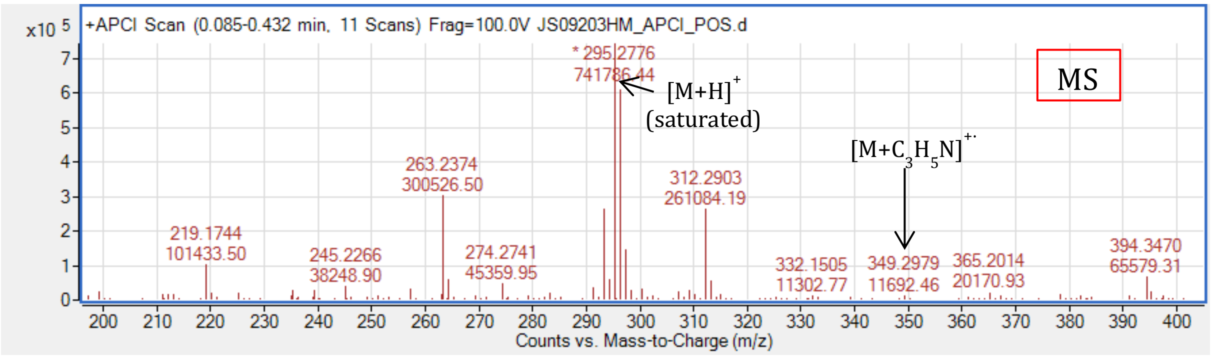


| Best | Formula | Score | Mass | ID Source | Diff (ppm) | Mass (MFG) | Diff (mDa) | DBE |
|---|---|---|---|---|---|---|---|---|
| ⦿ | C22 H39 N O2 | 96.64 | 349.2987 | MFG | -1.66 | 349.2981 | -0.58 | 4 |

$[M+C_3H_5N]^{+\cdot}$

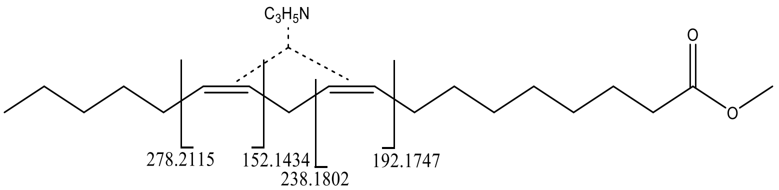


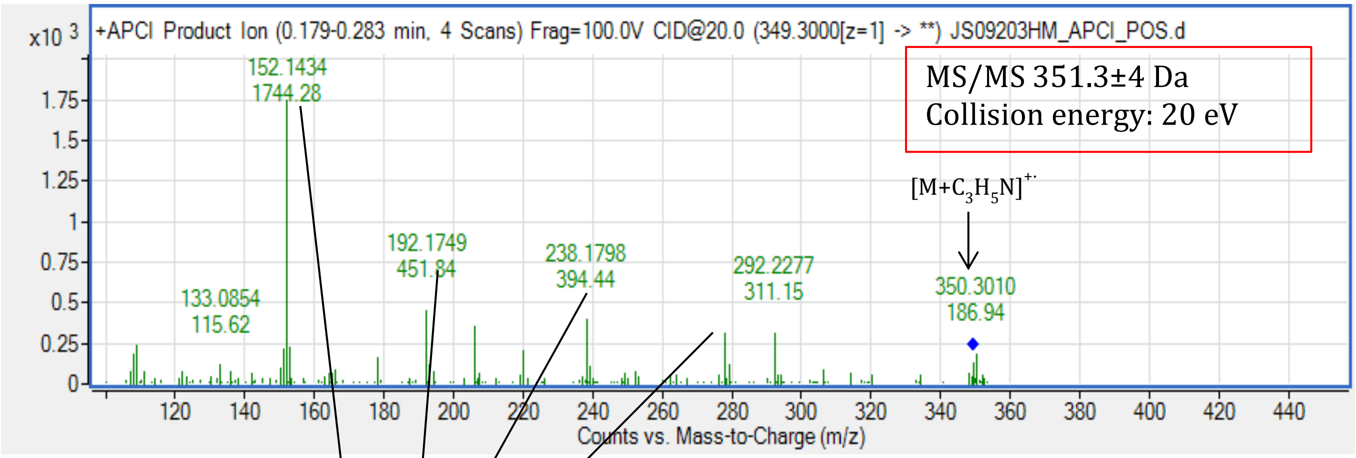


| Best | Formula | Score | Mass | ID Source | Diff (ppm) | Mass (MFG) | Diff (mDa) | DBE |
|---|---|---|---|---|---|---|---|---|
| ○ | C10 H18 N | 83.7 | 152.144 | MFG | -0.29 | 152.1439 | -0.04 | 2.5 |
| ⦿ | C13 H22 N | 87.62 | 192.1751 | MFG | 0.42 | 192.1752 | 0.08 | 3.5 |
| ○ | C14 H24 N O2 | 65.77 | 238.1808 | MFG | -0.52 | 238.1807 | -0.12 | 3.5 |
| ○ | C17 H28 N O2 | 75.37 | 278.2122 | MFG | -0.61 | 278.212 | -0.17 | 4.5 |

*Figure S32. HRMS analyses of **3***

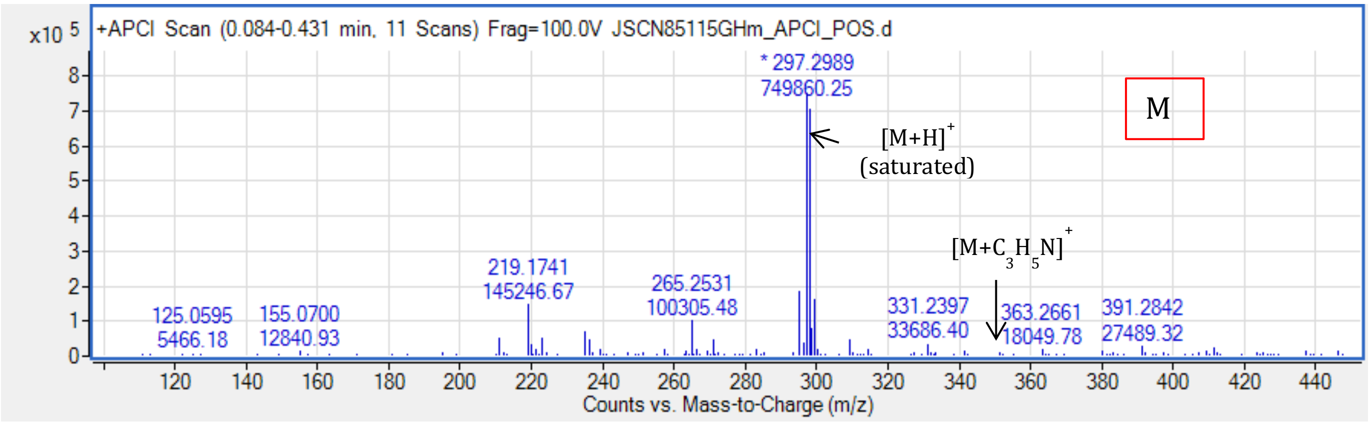


| Best | Formula | Score | Mass | ID Source | Diff (ppm) | Mass (MFG) | Diff (mDa) | DBE |
|---|---|---|---|---|---|---|---|---|
| ◉ | C22 H41 N O2 | 88.16 | 351.3139 | MFG | -0.46 | 351.3137 | -0.16 | 3 |

$[M+C_3H_5N]^{+\cdot}$

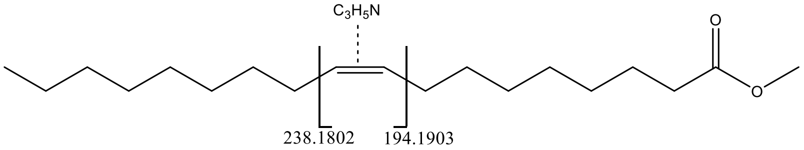


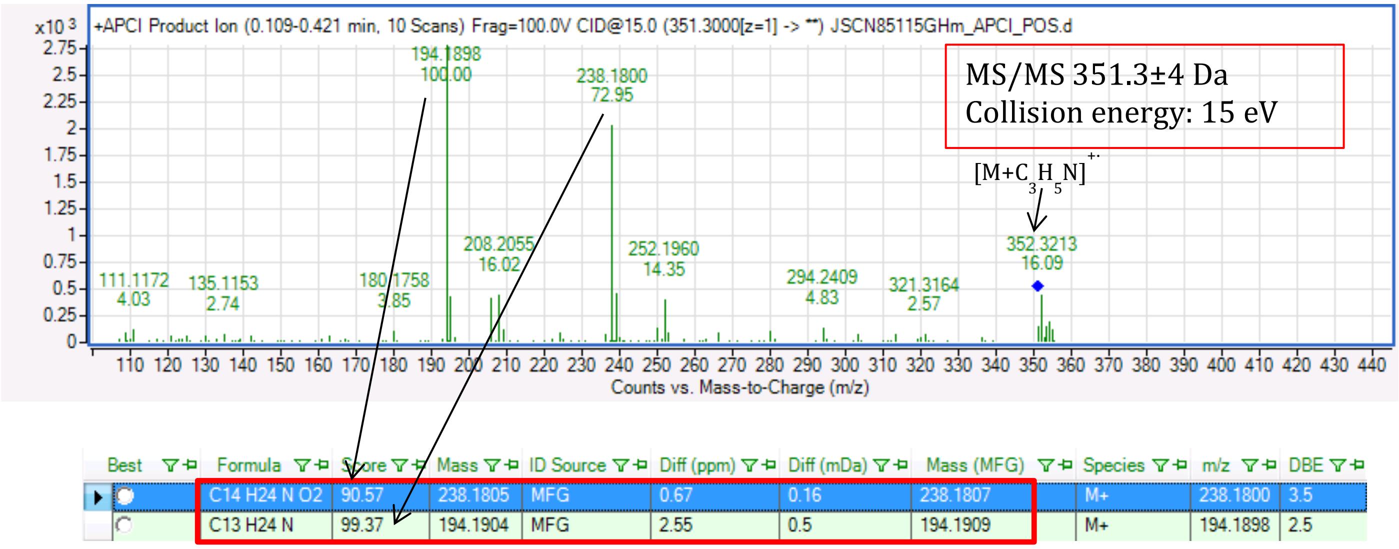


| Best | Formula | Score | Mass | ID Source | Diff (ppm) | Diff (mDa) | Mass (MFG) | Species | m/z | DBE |
|---|---|---|---|---|---|---|---|---|---|---|
| ○ | C14 H24 N O2 | 90.57 | 238.1805 | MFG | 0.67 | 0.16 | 238.1807 | M+ | 238.1800 | 3.5 |
| ○ | C13 H24 N | 99.37 | 194.1904 | MFG | 2.55 | 0.5 | 194.1909 | M+ | 194.1898 | 2.5 |

*Figure S33. CD spectra (MeOH) of ovalicin (**1**) and ovalicin esters (**2**–**4**).*

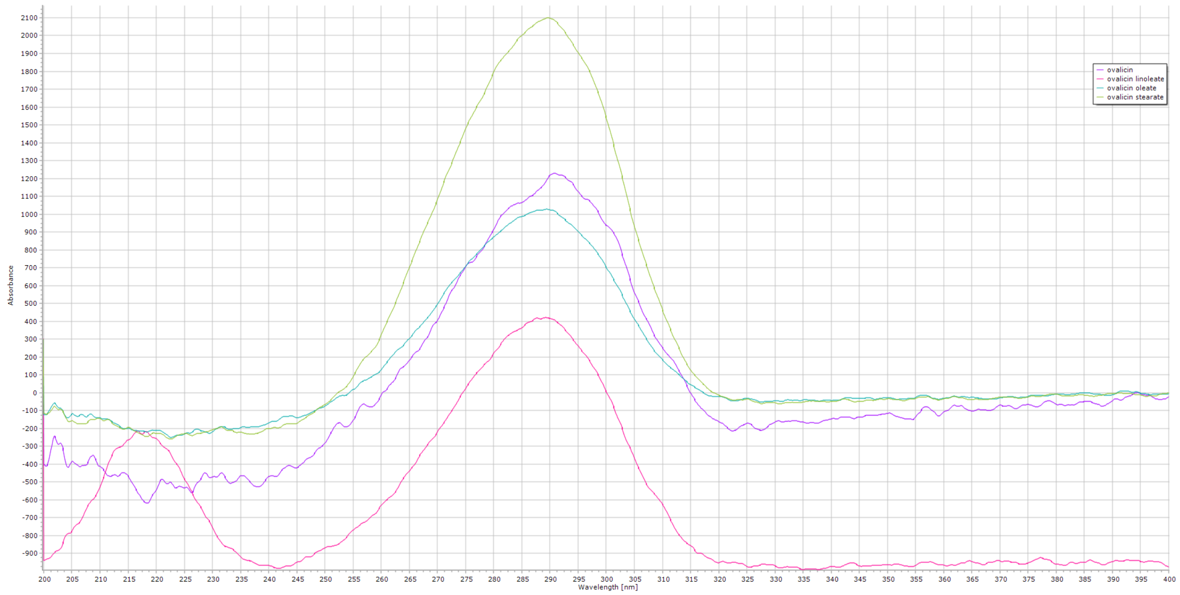


*Figure S34. Synthesis of hemisynthetic compounds **2b**, **3b** and **4b***

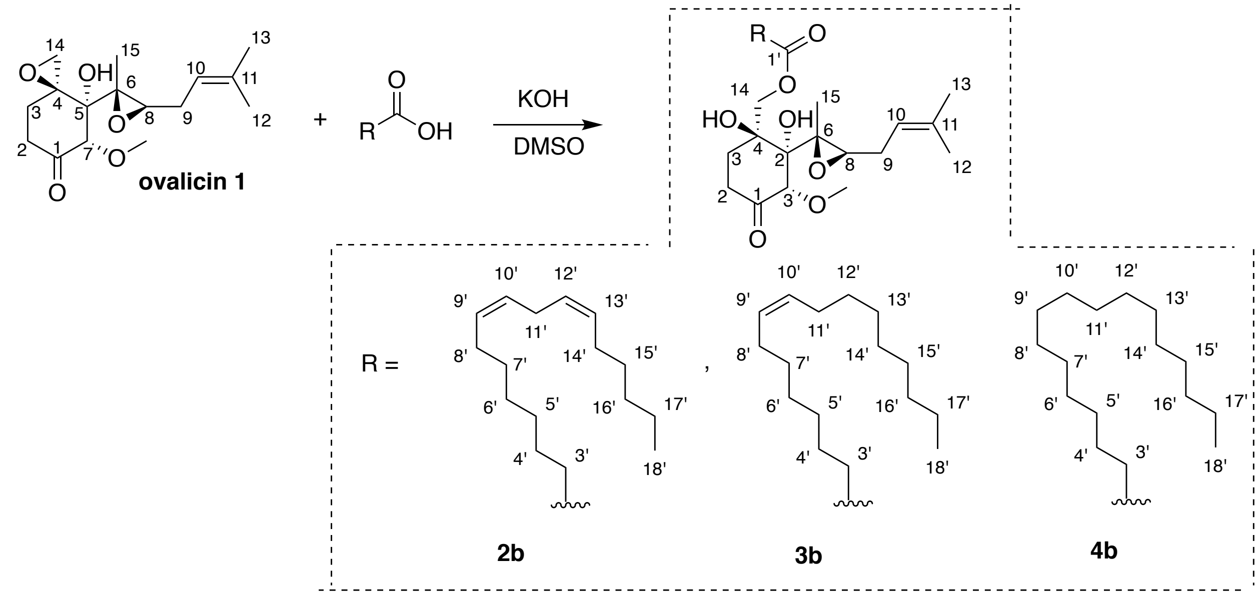

*Table S6. Stereochemistry of the fatty acid double bounds*

Reference [39]: Gunstone *et al.*, Chemistry and Physics of Lipids, 1977,18, 115-129

| | c9/c12 :C18 | c9/t12 :C18 | t9/c12 :C18 | t9/t12 :C18 | **2** |
|---|---|---|---|---|---|
| $\delta_C$ | | | | | |
| C8 | 27.3 | 27.2/32.6 | 27.2/32.6 | 32.5 | **28.3** |
| C9 | 130.01 | 130.27 | 130.64 | 130.92 | 131.0 |
| C10 | 128.24 | 127.96/128.59 | 127.96/128.59 | 128.79 | 129.3 |
| C11 | 25.77 | 30.56 | 30.56 | 35.68 | **26.7** |
| C12 | 128.08 | 128.45/127.83 | 128.45/127.83 | 128.65 | 129.2 |
| C13 | 130.20 | 130.79 | 130.41 | 131.08 | 131.1 |
| C14 | 27.32 | 32.67/27.22 | 32.67/27.22 | 35.59 | 28.3 |

Length of the fatty chain: C18; c : cis; t : trans

*Figure S35. $^{1}$H NMR (500 MHz, $CD_3OD$) spectrum of synthetic ovalicin linoleate (**2b**)*

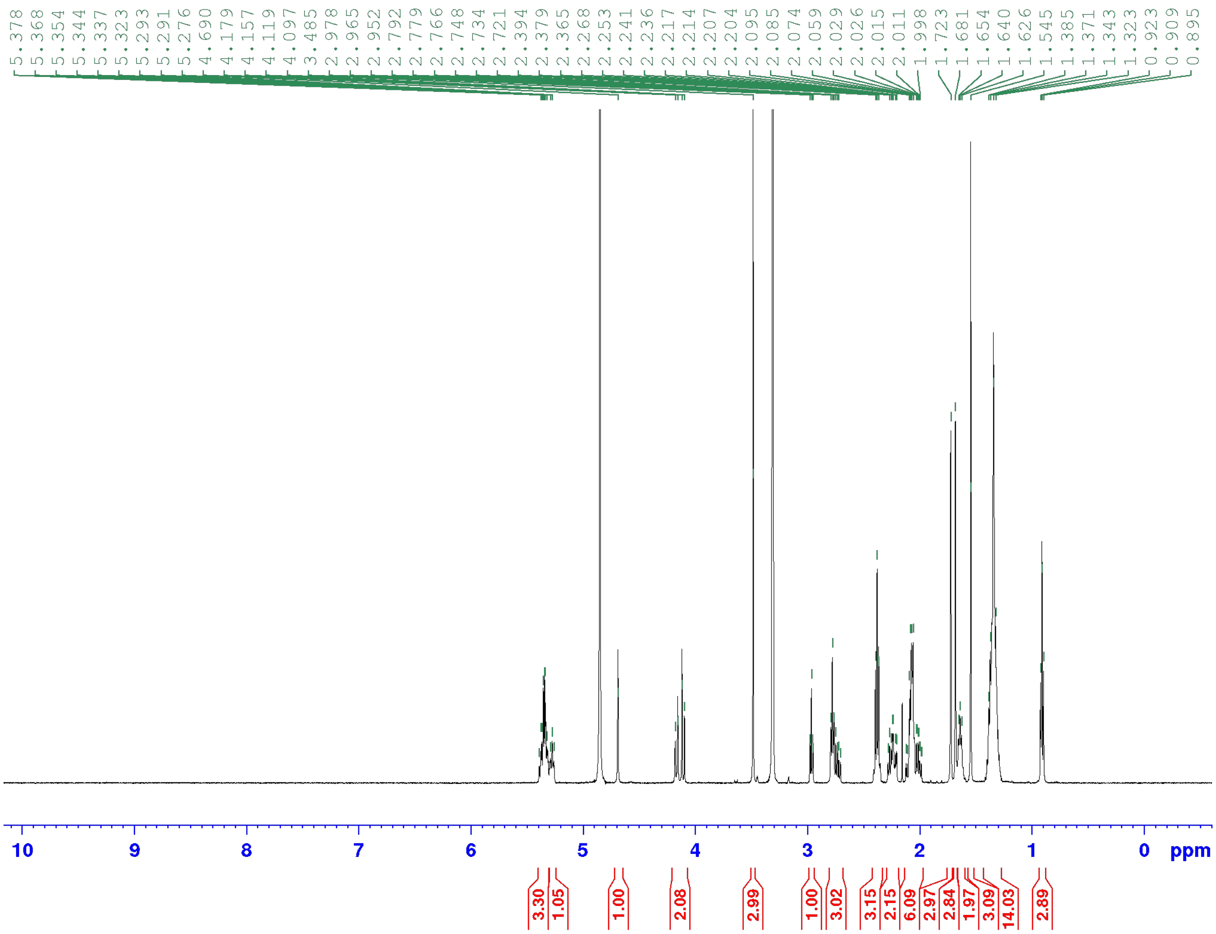


*Figure S36. Comparison between $^{1}$H NMR (500 MHz, $CD_3OD$) spectra of synthetic ovalicin linoleate (**2b**) (in red) and natural Ovalicin linoleate (**2**) (in black).*

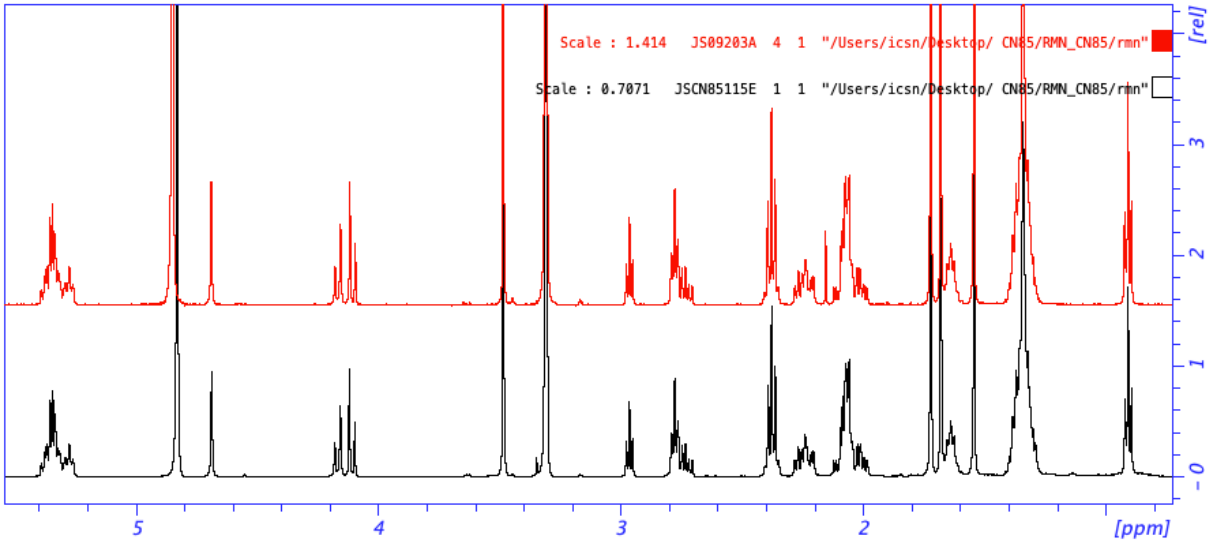


*Figure S37*. $^{1}H$ NMR (500 MHz, $CD_3OD$) spectrum of synthetic ovalicin oleate (**3b**)

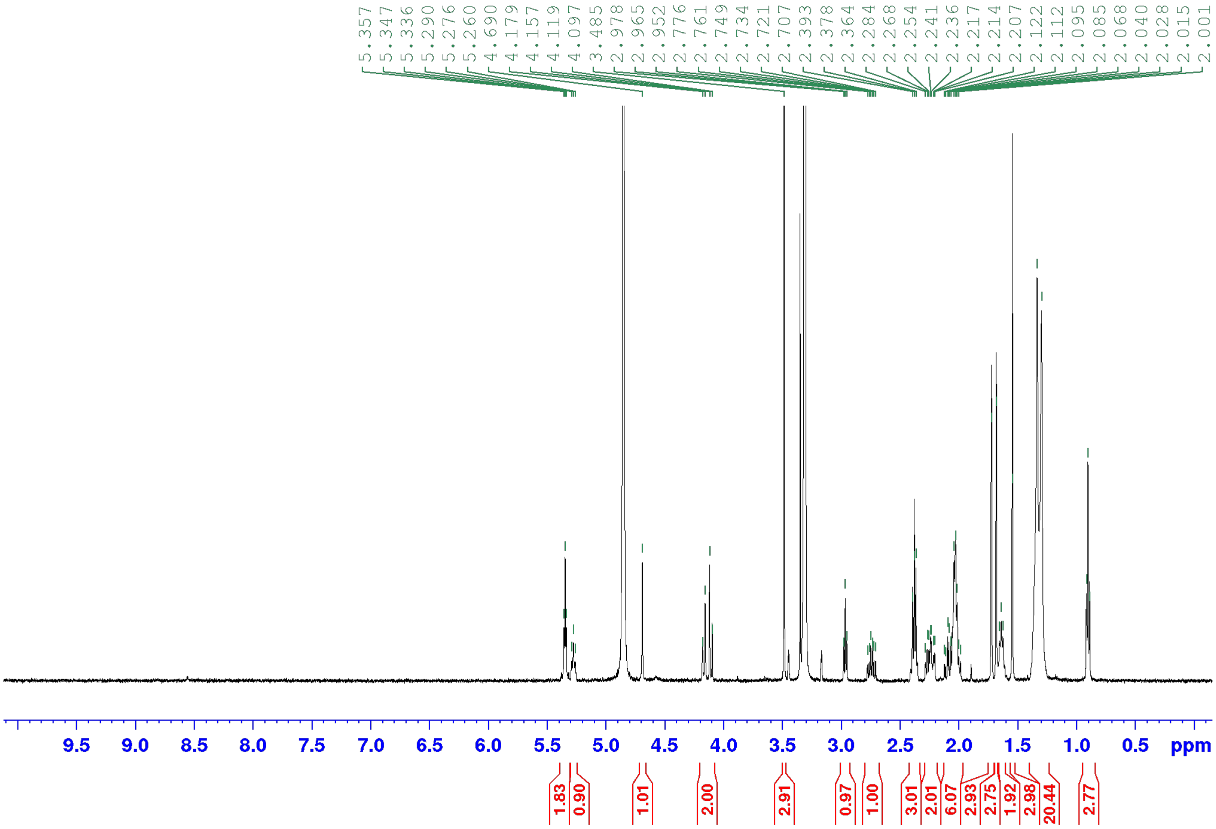

*Figure S38. Comparison between $^1$H NMR (500 MHz, $CD_3OD$) spectra of synthetic ovalicin oleate (**3b**) (in red) and natural ovalicin oleate (**3**) (in black).*

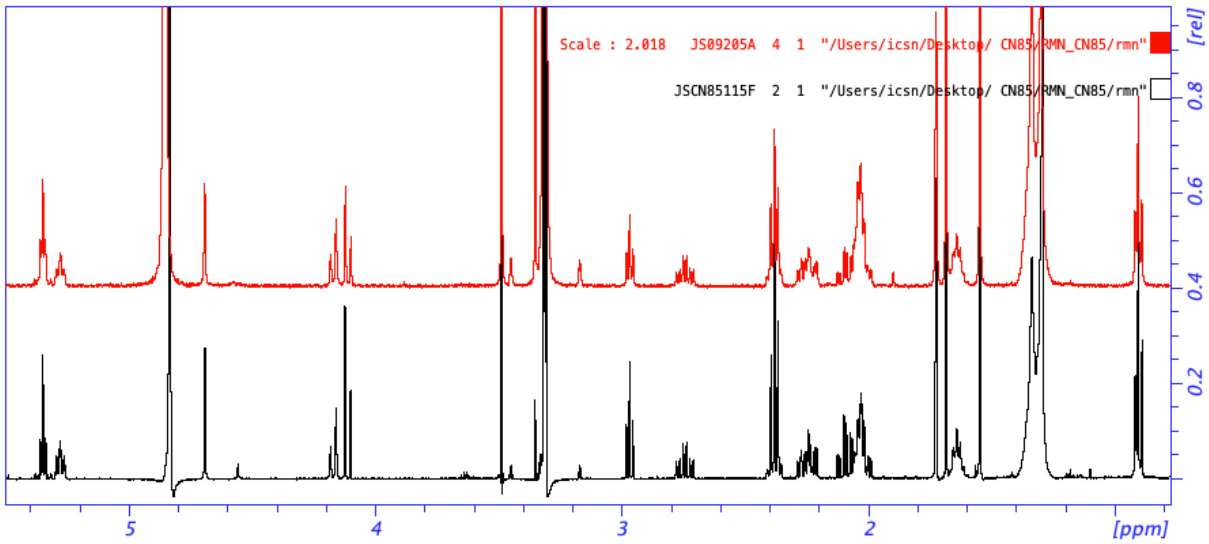

*Figure S39.* *¹H NMR (500 MHz, $CD_3OD$) spectrum of synthetic ovalicin stearate (**4b**)*

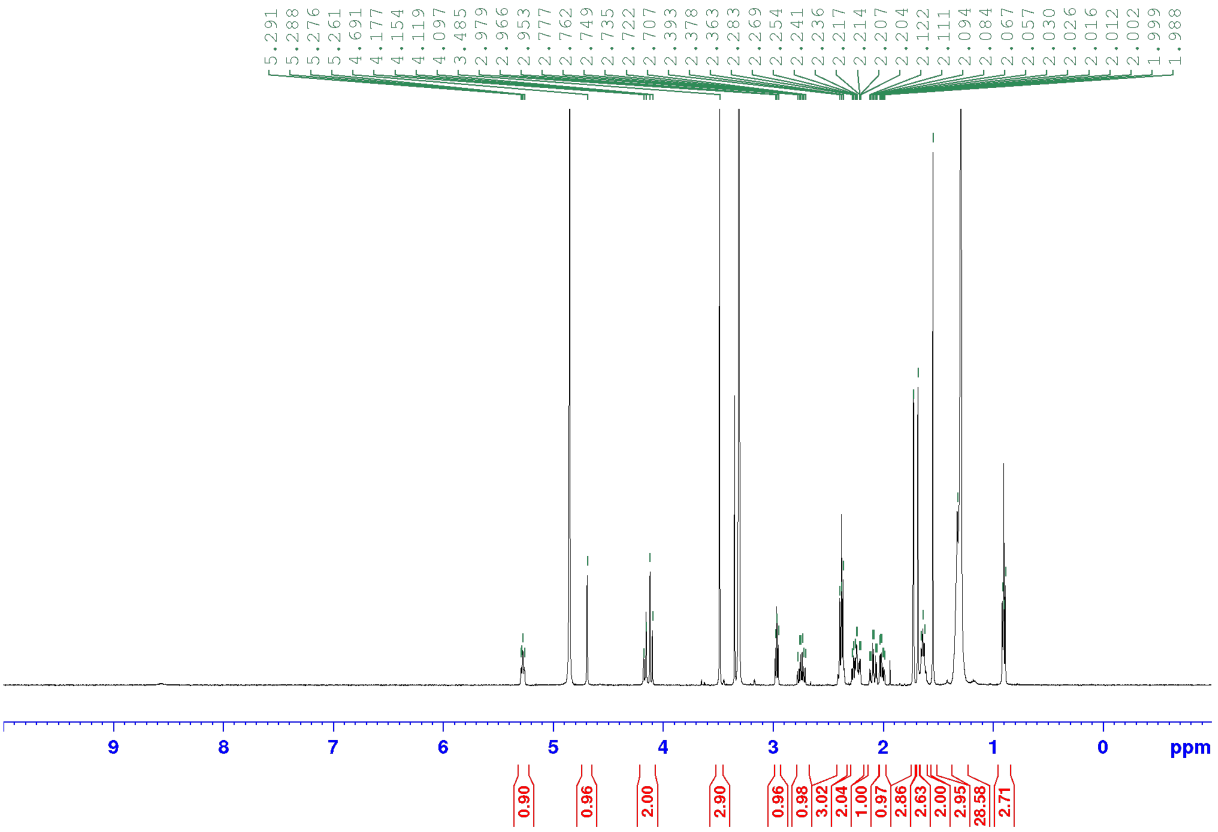


*Figure S40.* *Comparison between ¹H NMR (500 MHz, $CD_3OD$) spectra of synthetic ovalicin stearate (**4b**) (in red) and natural ovalicin stearate (**4**) (in black).*

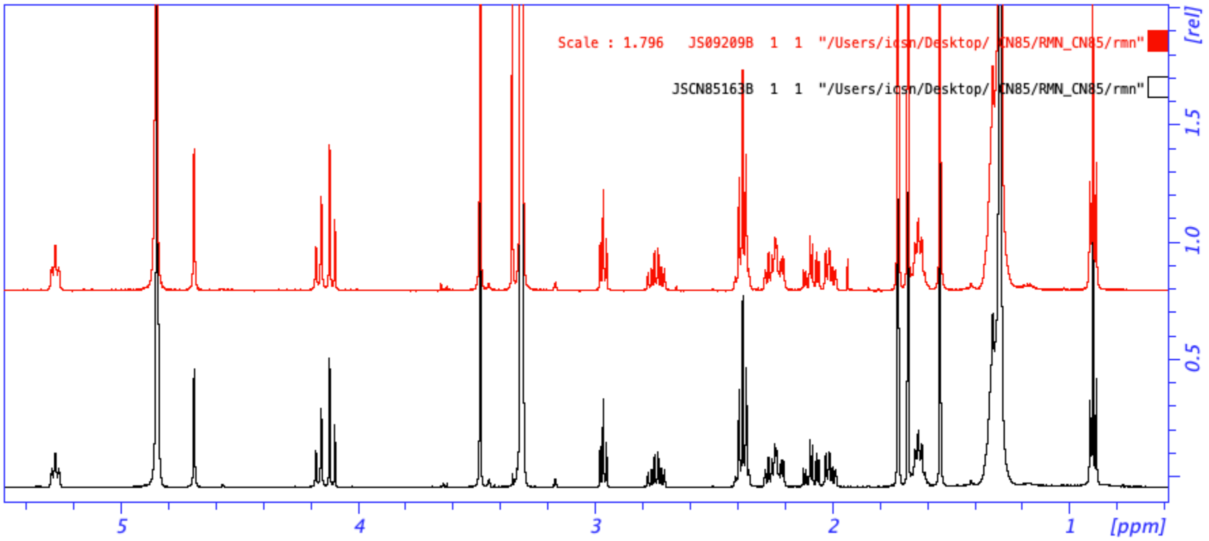

*Figure S41*. *ESI-HRMS spectra of* ***2b***

**Single Mass Analysis**
Tolerance = 5.0 PPM / DBE: min = -1.5, max = 100.0
Element prediction: Off
Number of isotope peaks used for i-FIT = 9
Monoisotopic Mass, Even Electron Ions
505 formula(e) evaluated with 4 results within limits (all results (up to 1000) for each mass)
Elements Used:

| Mass | Calc. Mass | mDa | PPM | DBE | Formula | i-FIT | i-FIT (Norm) | C | H | N | O | Na |
|---|---|---|---|---|---|---|---|---|---|---|---|---|
| 599.3911 | 599.3924 | -1.3 | -2.2 | 6.5 | C34 H56 O7 Na | 447.8 | 0.5 | 34 | 56 | | 7 | 1 |
| | 599.3884 | 2.7 | 4.5 | 2.5 | C29 H56 N2 O9 Na | 448.8 | 1.4 | 29 | 56 | 2 | 9 | 1 |
| | 599.3897 | 1.4 | 2.3 | 7.5 | C30 H52 N6 O5 Na | 449.9 | 2.5 | 30 | 52 | 6 | 5 | 1 |
| | 599.3937 | -2.6 | -4.3 | 11.5 | C35 H52 N4 O3 Na | 450.6 | 3.2 | 35 | 52 | 4 | 3 | 1 |

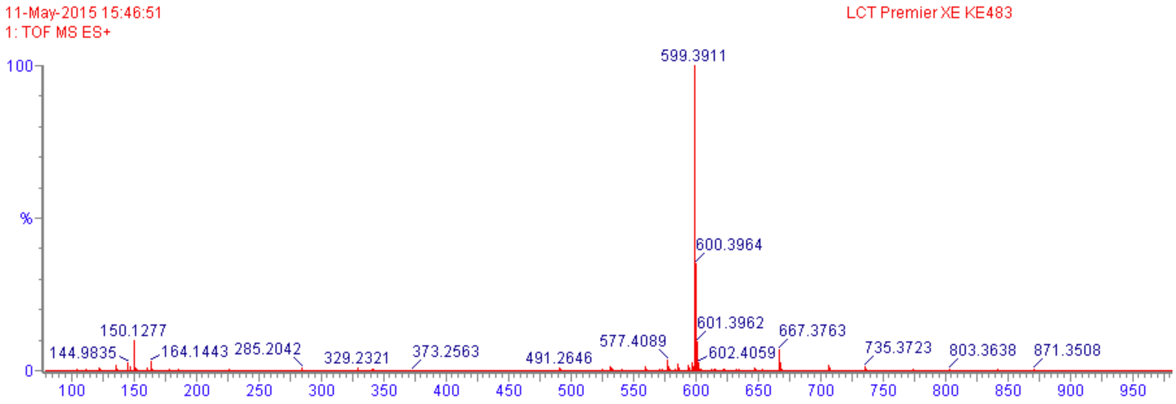


*Figure S42*. *ESI-HRMS spectra of* ***3b***

**Single Mass Analysis**
Tolerance = 5.0 PPM / DBE: min = -1.5, max = 100.0
Element prediction: Off
Number of isotope peaks used for i-FIT = 9
Monoisotopic Mass, Even Electron Ions
502 formula(e) evaluated with 3 results within limits (all results (up to 1000) for each mass)
Elements Used:

| Mass | Calc. Mass | mDa | PPM | DBE | Formula | i-FIT | i-FIT (Norm) | C | H | N | O | Na |
|---|---|---|---|---|---|---|---|---|---|---|---|---|
| 601.4056 | 601.4080 | -2.4 | -4.0 | 5.5 | C34 H58 O7 Na | 420.9 | 0.0 | 34 | 58 | | 7 | 1 |
| | 601.4053 | 0.3 | 0.5 | 6.5 | C30 H54 N6 O5 Na | 426.3 | 5.4 | 30 | 54 | 6 | 5 | 1 |
| | 601.4040 | 1.6 | 2.7 | 1.5 | C29 H58 N2 O9 Na | 426.5 | 5.6 | 29 | 58 | 2 | 9 | 1 |

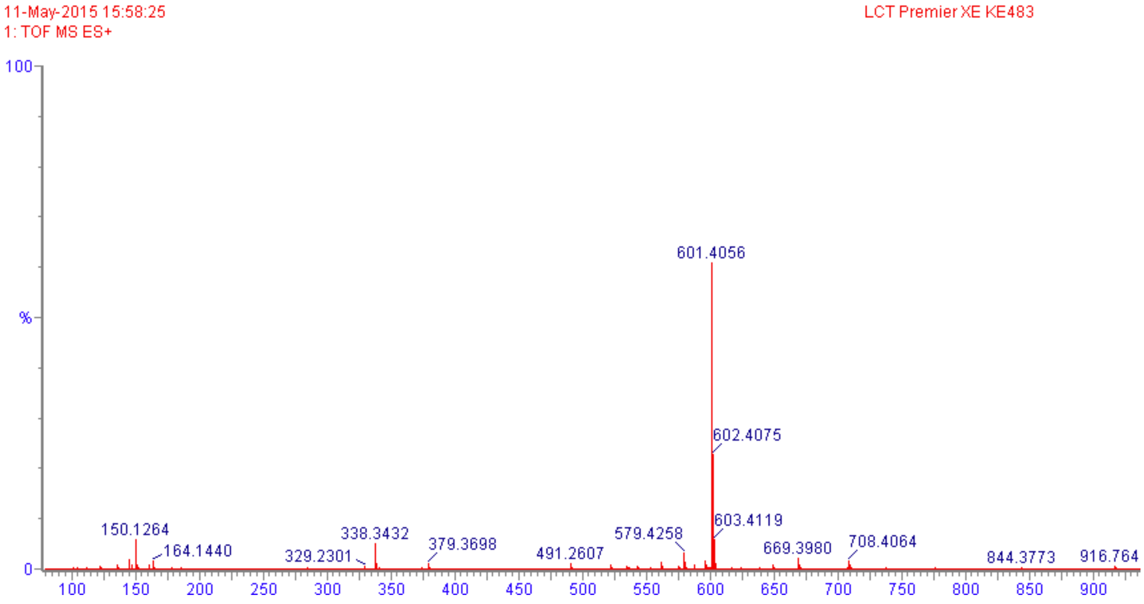

*Figure S43***.** *ESI-HRMS spectra of* ***4b***

**Single Mass Analysis**
Tolerance = 5.0 PPM / DBE: min = -1.5, max = 100.0
Element prediction: Off
Number of isotope peaks used for i-FIT = 9
Monoisotopic Mass, Even Electron Ions
503 formula(e) evaluated with 3 results within limits (all results (up to 1000) for each mass)
Elements Used:

| Mass | Calc. Mass | mDa | PPM | DBE | Formula | i-FIT | i-FIT (Norm) | C | H | N | O | Na |
|---|---|---|---|---|---|---|---|---|---|---|---|---|
| 603.4229 | 603.4237 | -0.8 | -1.3 | 4.5 | C34 H60 O7 Na | 413.7 | 0.1 | 34 | 60 | | 7 | 1 |
| | 603.4250 | -2.1 | -3.5 | 9.5 | C35 H56 N4 O3 Na | 415.8 | 2.2 | 35 | 56 | 4 | 3 | 1 |
| | 603.4210 | 1.9 | 3.1 | 5.5 | C30 H56 N6 O5 Na | 418.3 | 4.7 | 30 | 56 | 6 | 5 | 1 |

03-Jun-2015 4::6::1 LCT Premier XE KE483
1: TOF MS ES+

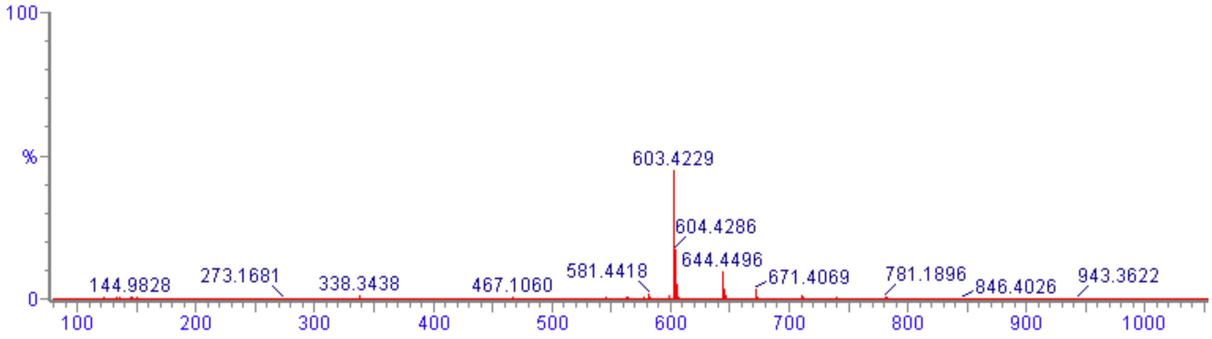

*Figure S44. (A) Molecular network of crude extracts of P. boydii. SNB-CN71, -CN73, -CN81, -CN85 (B) Cluster for the sodiated molecular ion of pseurotin A (C) Cluster containing the sodiated molecular ions of ovalicin (1) and its ester analogues. (D) Cluster of the protonated molecular ions of tyroscherin and N-methyltyrocherin. Relative quantification of each ion within the fractions are represented as a XIC area-dependent pie-chart drawing.*

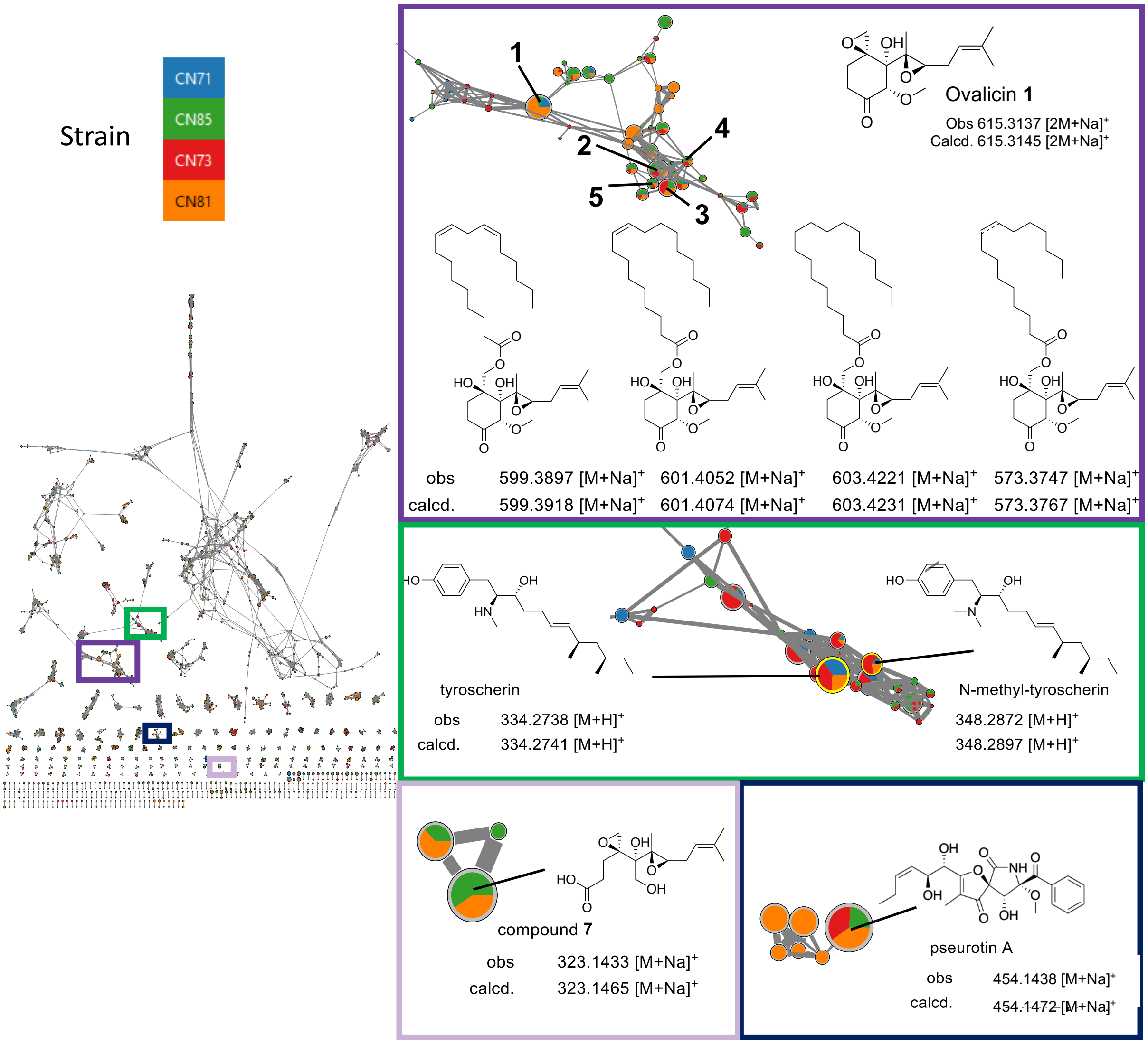

*Figure S45. fragmentation spectra of ovalicin fatty acids*

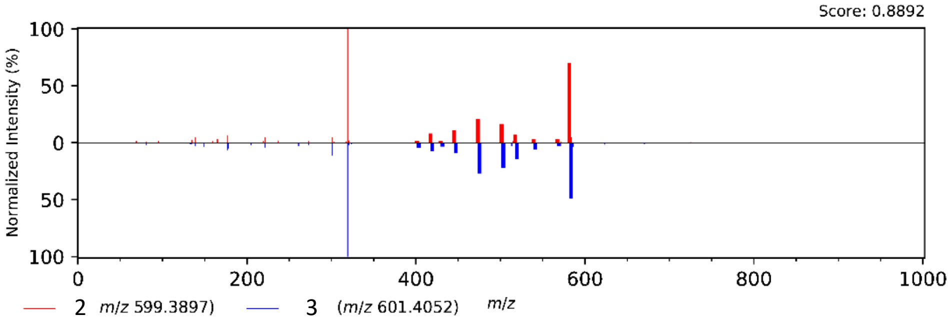


Main common fragment: 319.1518 [Ovalicine + Na]$^{+}$
10 common neutral losses, all respective fragment with *m/z* difference of 2.0169 ($H_2$) specifics to unsaturation

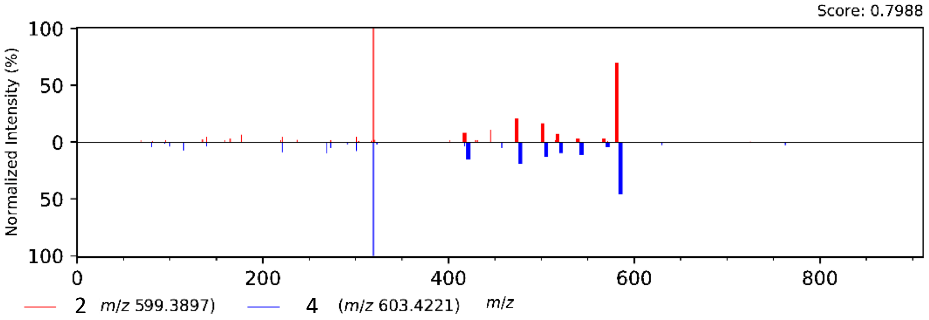


Main common fragment: 319.1518 [Ovalicine + Na]$^{+}$
8 common neutral losses, all respective fragments with *m/z* difference of 4.0248 ($H_4$) specifics to 2 unsaturation

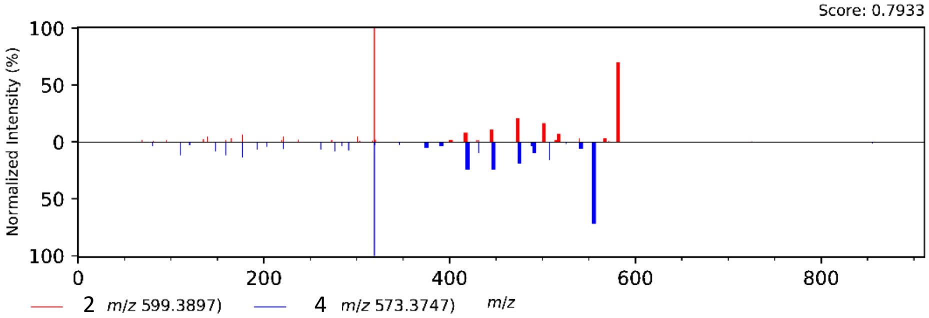


Main common fragment: 319.1518 [Ovalicine + Na]$^{+}$
9 common neutral losses, all respective fragments with *m/z* difference of 26.0172 ($C_2H_2$)

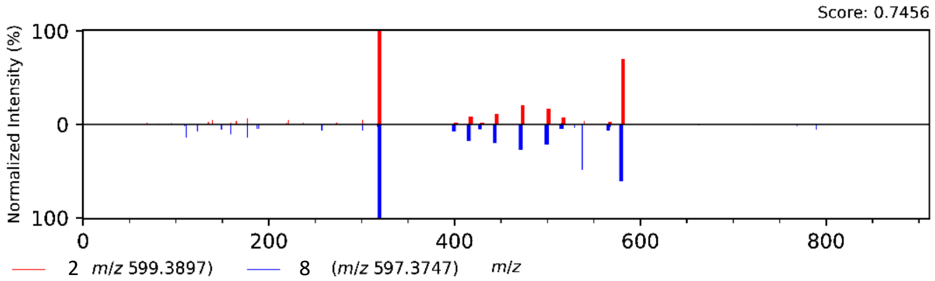


Main common fragment: 319.1518 [Ovalicine + Na]$^+$
9 common neutral losses, all respective fragments with *m/z* difference of 2.0169 ($H_2$) specifics to 1 unsaturation

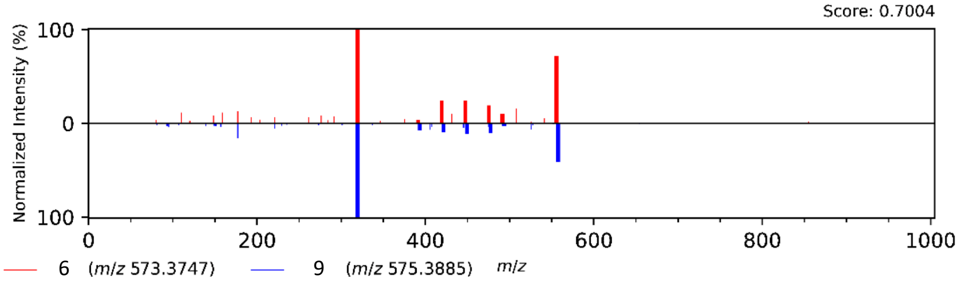


Main common fragment: 319.1518 [Ovalicine + Na]$^+$
6 common neutral losses, all respective fragments with *m/z* difference of 2.0169 ($H_2$) specifics to 1 unsaturation

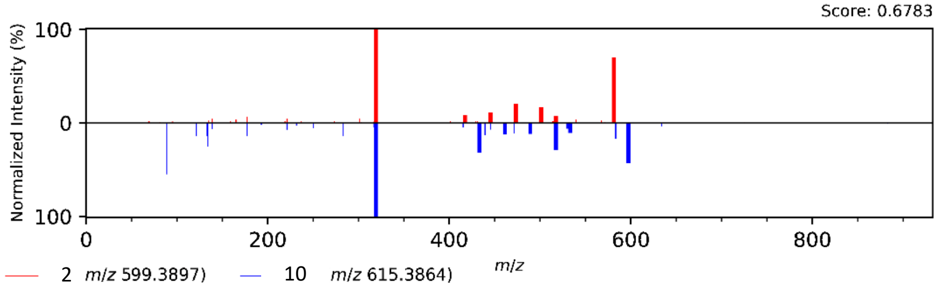


Main common fragment: 319.1518 [Ovalicine + Na]$^+$
6 common neutral losses, all respective fragments with *m/z* difference of 15.9967 (O) specifics unsaturation epoxidation

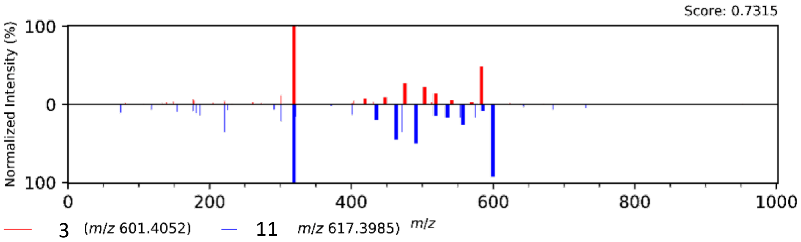


Main common fragment: 319.1518 [Ovalicine + Na]+

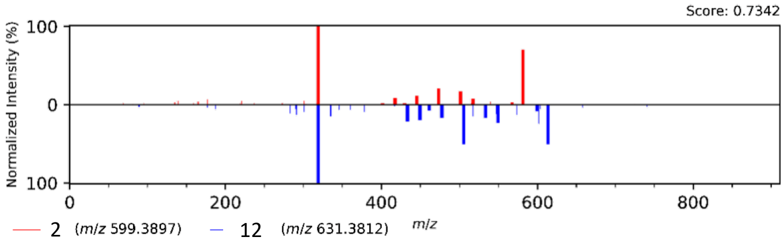


Main common fragment: 319.1518 [Ovalicine + Na]+
7 common neutral losses, all respective fragments with *m/z* difference of 31.9915 ($O_2$) specifics to 2 unsaturation epoxidations

*Table S7. Basic genomes statistics and BUSCO scores*

| ***P. boydii* strain** | **Genome size (Mb)** | **Contigs** | **N50 (Mb)** | **Busco score (%)** | **Transcripts number** |
|---|---|---|---|---|---|
| CN71 | 43 | 18 | 5.3 | 96.1 | 10 810 |
| CN73 | 42 | 27 | 3.3 | 96 | 10 815 |
| CN81 | 42 | 57 | 1.6 | 95.9 | 10 821 |
| CN85 | 42 | 36 | 2.1 | 96 | 10 802 |

*Table S8. Pbo_Ova biosynthetic gene cluster annotation*

| Gene name | | Size (AA) | Best match Swissprot | Global alignement (I/S/G) % | Global alignement (I/S/G) AA | Domaine_Pfam | PFAM_ID | Plausible_function | Duplicated |
|---|---|---|---|---|---|---|---|---|---|
| *Pbo_Ova_A* | CN71 | 94 | M4VQY9 | 22/25/68 | 63/72/195 | UbiA prenyltransferase family | PF01040 | Terpene cyclase | N |
| | CN73 | 94 | | 22/25/68 | 63/72/195 | | | | |
| | CN81 | 105 | | 23/26/65 | 66/77/190 | | | | |
| | CN85 | 182 | | 26/31/59 | 87/104/197 | | | | |
| *Pbo_Ova_B* | CN71 | 536 | Q4WAZ6 | 74/85/1 | 396/456/4 | Cytochrome P450 | PF00067 | Cytochrome P450 monooxygenase | N |
| | CN73 | | | 74/85/1 | 396/456/4 | | | | |
| | CN81 | | | 73/84/1 | 395/454/4 | | | | |
| | CN85 | | | 74/85/1 | 396/456/4 | | | | |
| *Pbo_Ova_C* | CN71 | 272 | Q4WAZ3 | 64/75/13 | 199/232/39 | Phytanoyl-CoA dioxygenase | PF05721 | dioxygenase | N |
| | CN73 | | | 64/75/13 | 199/232/39 | | | | |
| | CN81 | | | 64/75/13 | 197/231/39 | | | | |
| | CN85 | | | 64/75/13 | 201/233/39 | | | | |
| *Pbo_Ova_D* | CN71 | 396 | A0A067Z9B6 | 71/84/1 | 283/337/4 | O-methyltransferase | PF00891 | O-methyltransferase | N |
| | CN73 | 396 | | 71/84/1 | 283/337/4 | | | | |
| | CN81 | 396 | | 71/85/1 | 285/339/4 | | | | |
| | CN85 | 400 | | 71/84/1 | 283/337/4 | | | | |
| *Pbo_Ova_E* | CN71 | 160 | Q0D155 | 40/59/7 | 66/98/12 | EthD | PF07110 | Oxydoreductase | N |
| | CN73 | | | 40/59/7 | 66/98/12 | | | | |
| | CN81 | | | 40/61/7 | 66/101/12 | | | | |
| | CN85 | | | 40/59/7 | 66/99/12 | | | | |
| *Pbo_Ova_F* | CN71 | 310 | D4AU26 | 49/67/1 | 152/209/3 | Phytanoyl-CoA dioxygenase | PF05721 | dioxygenase | N |
| | CN73 | | | 49/67/1 | 152/209/3 | | | | |
| | CN81 | | | 49/67/1 | 152/208/3 | | | | |
| | CN85 | | | 49/67/1 | 152/209/3 | | | | |
| *Pbo_Ova_G* | CN71 | 394 | Q9BV23 | 17/30/41 | 79/136/189 | Alpha/beta hydrolase | PF12697 | hydrolase | N |
| | CN73 | | | 17/30/41 | 79/136/189 | | | | |
| | CN81 | | | 17/30/41 | 78/136/189 | | | | |
| | CN85 | | | 17/30/41 | 79/136/189 | | | | |
| *Pbo_Ova_H* | CN71 | 584 | P84285 | 33/52/18 | 213/338/115 | membrane-bound O-acyltransferase | PF03062 | Acyltransferase | N |
| | CN73 | 584 | | 33/52/18 | 213/338/115 | | | | |
| | CN81 | 547 | | 29/46/23 | 192/306/152 | | | | |
| | CN85 | 584 | | 33/51/18 | 213/336/119 | | | | |

*Table S9. Pbo_Pse biosynthetic gene cluster annotation*

| Gene name | | Size (AA) | Best match Swissprot | Global alignement (I/S/G) % | Global alignement (I/S/G) AA | Domaine_Pfam | PFAM_ID | Plausible_function | Duplicated |
|---|---|---|---|---|---|---|---|---|---|
| *Pbo_Pse_A* | CN71 | 4065 | Q4WAZ9 | 62/77/3 | 2544/3147/118 | Beta-ketoacyl synthase<br>Acyl transferase<br>Polyketide synthase dehydratase<br>Ketoreductase<br>Condensation<br>Ketoreductase<br>Male sterility | PF00109<br>PF00698<br>PF14765<br>PF08659<br>PF00668<br>PF08659<br>PF07993 | PKS-NRPS hybrid synthetase | N |
| | CN73 | | | 62/77/3 | 2544/3147/118 | | | | |
| | CN81 | | | 62/77/2 | 2541/3144/96 | | | | |
| | CN85 | | | 62/77/3 | 2549/3145/118 | | | | |
| *Pbo_Pse_B* | CN71 | 900 | Q4WAZ0 | 62/77/2 | 562/702/17 | Flavin-binding monooxygenase<br>Methyltransferase | PF00743<br>PF08242 | Dual-functional monooxygenase/ methyltransferase | N |
| | CN73 | 900 | | 62/77/2 | 562/702/17 | | | | |
| | CN81 | 908 | | 63/78/1 | 573/708/11 | | | | |
| | CN85 | 900 | | 62/77/2 | 562/704/17 | | | | |
| *Pbo_Pse_C* | CN71 | 445 | Q4WB01 | 68/77/14 | 351/396/73 | Cytochrome P450 | PF00067 | Cytochrome P450 monooxygenase | N |
| | CN73 | | | 68/77/14 | 351/396/73 | | | | |
| | CN81 | | | 68/76/14 | 350/395/73 | | | | |
| | CN85 | | | 68/77/14 | 351/396/73 | | | | |
| *Pbo_Pse_D* | CN71 | 271 | Q4WB00 | 68/82/0 | 185/223/0 | Methyltransferase | PF13489 | Methyltransferase | N |
| | CN73 | | | 68/82/0 | 185/223/0 | | | | |
| | CN81 | | | 68/82/0 | 184/222/0 | | | | |
| | CN85 | | | 69/82/0 | 186/223/0 | | | | |
| *Pbo_Pse_E* | CN71 | 227 | Q4WB03 | 58/73/4 | 137/174/10 | Glutathione S-transferase | PF00043 | Glutathione S-transferase | N |
| | CN73 | | | 58/73/4 | 137/174/10 | | | | |
| | CN81 | | | 57/74/6 | 136/176/14 | | | | |
| | CN85 | | | 57/73/4 | 136/173/10 | | | | |
| *Pbo_Pse_F* | CN71 | 446 | Q4WAZ8 | 82/91/0 | 364/406/1 | Alpha/beta hydrolase | PF12697 | Hydrolase | N |
| | CN73 | | | 82/91/0 | 364/406/1 | | | | |
| | CN81 | | | 81/91/0 | 362/406/1 | | | | |
| | CN85 | | | 82/91/0 | 364/406/1 | | | | |

*Table S10. Annotated ovalicins from P. boydii crude extracts, synthetized by Pbo_Ova biosynthetic pathway.*

| ID | *m/z* | Error (ppm) | Species | Formula | Annotation Level |
|---|---|---|---|---|---|
| **Farnesyl pyrophosphate** | | | | $C_{15}H_{28}O_7P_2$ | |
| **β-trans-bergamoten** | | | | $C_{15}H_{24}$ | |
| **Btb1** | 251.1634 | 3.1 | $[M+H]^+$ | $C_{15}H_{22}O_3$ | 3 |
| **Btb2** | 267.1587 | 1.5 | $[M+H]^+$ | $C_{15}H_{22}O_4$ | 3 |
| **Btb3** | 303.1564 | 1.0 | $[M+Na]^+$ | $C_{16}H_{24}O_4$ | 3 |
| **1** | 319.1512 | 1.3 | $[M+Na]^+$ | $C_{16}H_{24}O_5$ | 0 |
| **Dihydro-ovalicin** | 321.1669 | -1.2 | $[M+Na]^+$ | $C_{16}H_{26}O_5$ | 2 |

Level 0 corresponds annotations from isolated pure compounds; level 1 to annotations by comparison with standards; level 2 to putative annotations (e.g., MS/MS library comparison or tentative structure); level 3 to a chemical class assignment

*Figure S46. Compounds **1** and dihydro-ovalicin MS/MS spectra with their main fragments in bold and common neutral loss in black.*

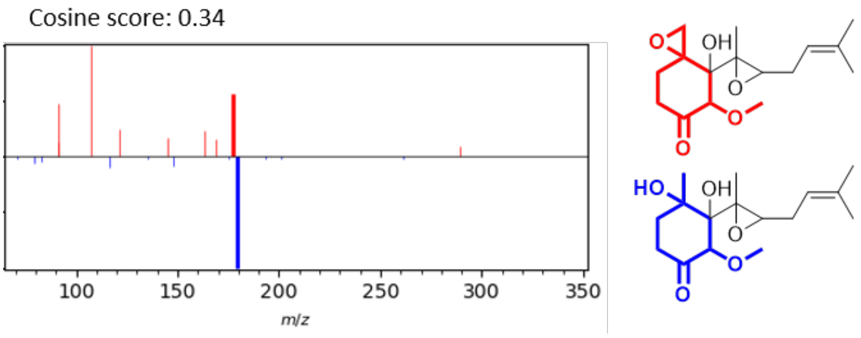

*Figure S47*. ¹H NMR (800 MHz, $CD_3OD$) spectrum of **7**

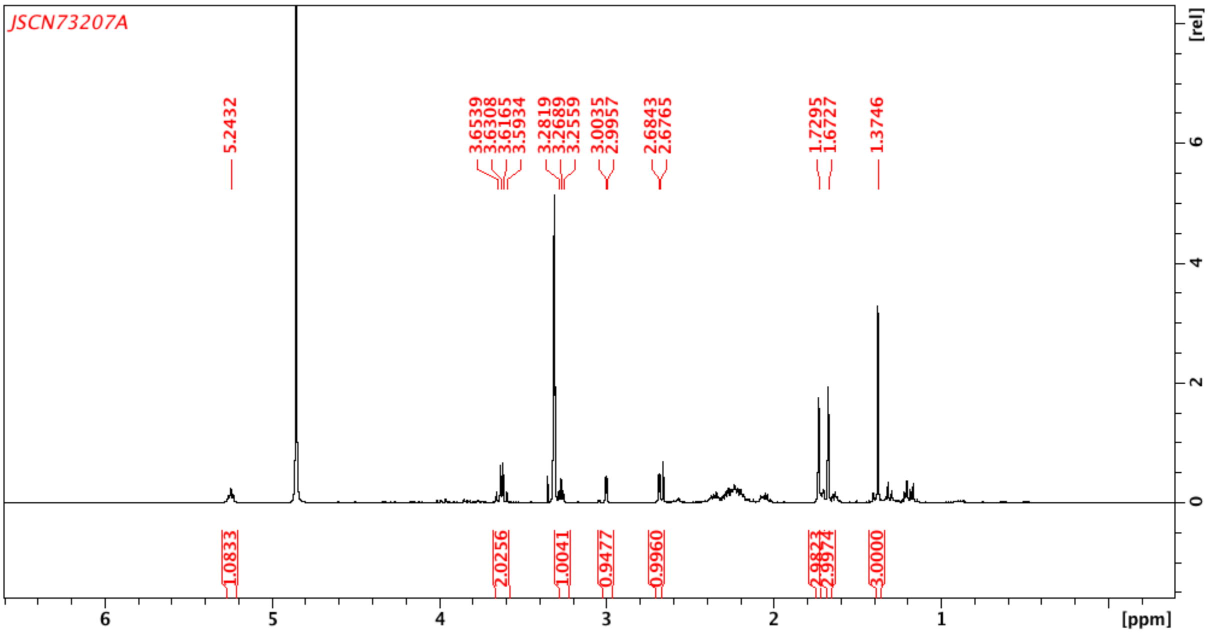


*Figure S48*. COSY (800 MHz, $CD_3OD$) spectrum **7**

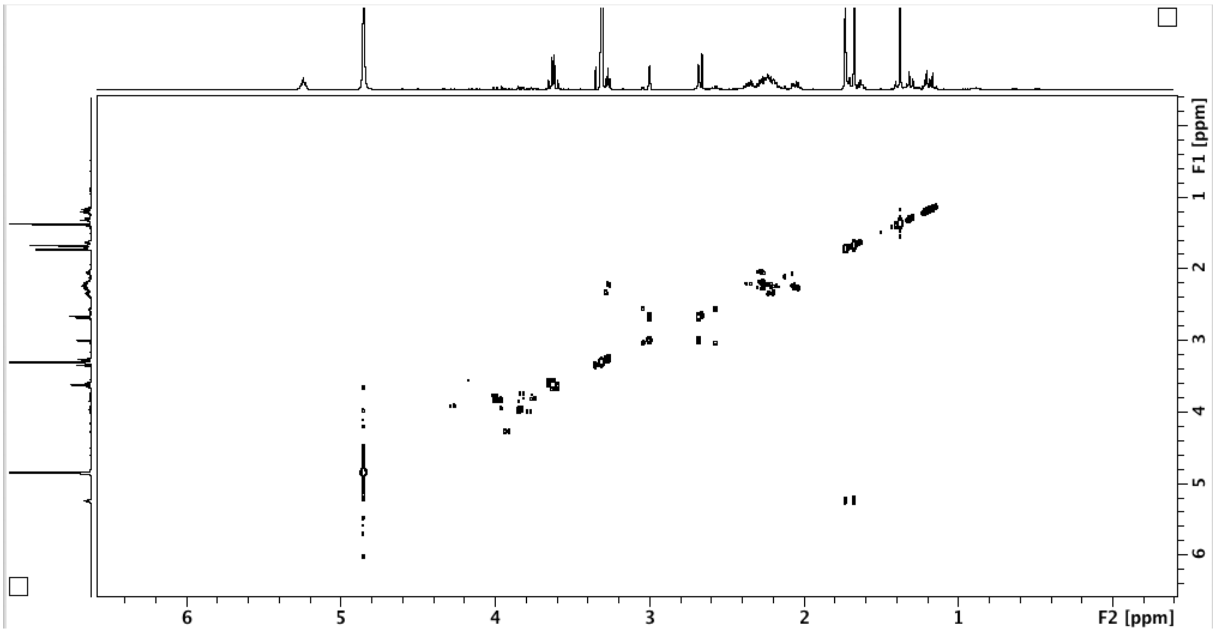

*Figure S49. HSQC (800 MHz, $CD_3OD$) spectrum of **7***

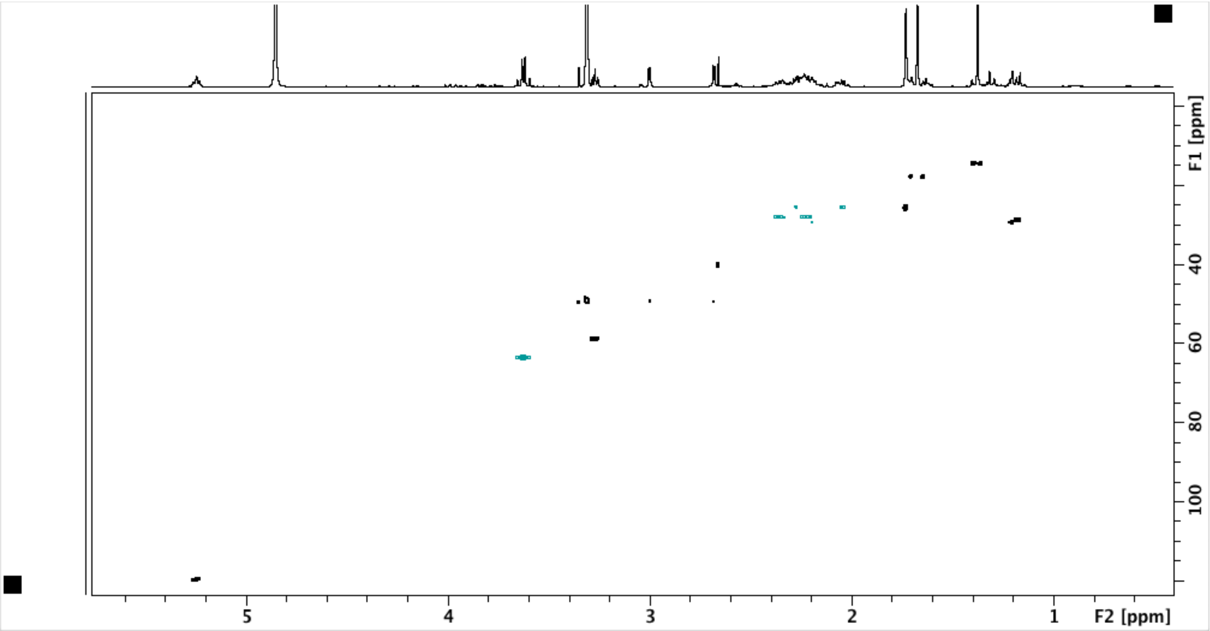


*Figure S50. HMBC (800 MHz, $CD_3OD$) spectrum of **7***

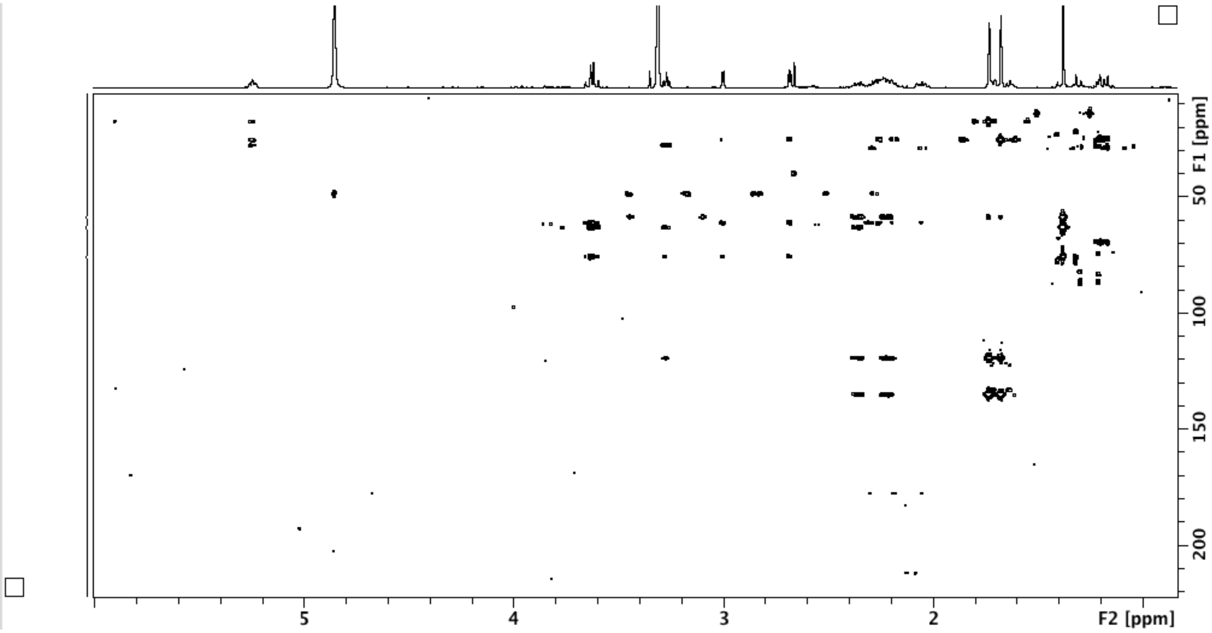

*Figure S51. ESI-HRMS of compound **7***

**Elemental Composition Report** — **Page 1 of 1**

**Single Mass Analysis**
Tolerance = 5.0 PPM / DBE: min = -1.5, max = 100.0
Element prediction: Off
Number of isotope peaks used for i-FIT = 9

Monoisotopic Mass, Even Electron Ions
420 formula(e) evaluated with 2 results within limits (all results (up to 1000) for each mass)
Elements Used:
C: 1-120 H: 1-150 N: 0-10 O: 0-15
STIEN_lsorres57-1 336 (1.529) Cm (333:341)
1: TOF MS ES+

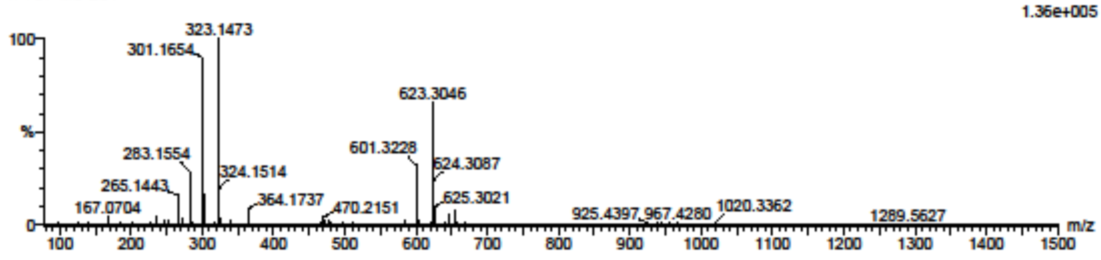


| Mass | Calc. Mass | mDa | PPM | DBE | i-FIT | i-FIT (Norm) | Formula |
|---|---|---|---|---|---|---|---|
| Minimum: | | | | -1.5 | | | |
| Maximum: | | 5.0 | 5.0 | 100.0 | | | |
| 301.1654 | 301.1651 | 0.3 | 1.0 | 3.5 | 1262.4 | 0.1 | C15 H25 O6 |
| | 301.1665 | -1.1 | -3.7 | 8.5 | 1265.2 | 2.9 | C16 H21 N4 O2 |

*Table S11. Full NMR data of 7 recorded in $CD_3OD$*

| Position | $\delta_C$, type | $\delta_H$ ($J$ in Hz) | COSY | HMBC |
|---|---|---|---|---|
| 1 | 178.0, C | | | |
| 2 | 29.3, $CH_2$ | 2.22 | H3b, H3a | C1, C3 |
| 3 | 25.6, $CH_2$ | (a) 2.27, m | H3b, H2 | |
| | | (b) 2.05, m | H3a, H2 | C1 |
| 4 | 61.5, C | | | |
| 5 | 76.0, C | | | |
| 6 | 63.4, C | | | |
| 7 | 63.6, $CH_2$ | (a) 3.64, d (11.6) | H15b | C6, C4, C5 |
| | | (b) 3.60, d (11.6) | H15a | C6, C4, C5 |
| 8 | 58.3, CH | 3.26, t (6.5) | H8 | C5, C6, C9, C10 |
| 9 | 28.0, $CH_2$ | (a) 2.36, m | H9 | C7, C9, C11, C12 |
| | | (b) 2.22, m | H4’ | C7, C10, C12, C13 |
| 10 | 119.8, C | 5.24, m | H9 | C9, C12, C13 |
| 11 | 135.4, C | | | |
| 12 | 25.8, $CH_3$ | 1.67, s | | C10, C11, C12 |
| 13 | 17.9, $CH_3$ | 1.73, s | | C10, C11, C12 |
| 14 | 48.8 | (a) 3.00, d (3.9) | H14b | C3, C4, C5 |
| | | (b) 2.68, d (3.9) | H14a | C3, C4, C5 |
| 15 | 17.6, $CH_3$ | 1.37, s | | C8, C5 |
